# Rostral growth of commissural axons requires the cell adhesion molecule MDGA2

**DOI:** 10.1186/1749-8104-6-22

**Published:** 2011-05-04

**Authors:** Pascal Joset, Andrin Wacker, Régis Babey, Esther A Ingold, Irwin Andermatt, Esther T Stoeckli, Matthias Gesemann

**Affiliations:** 1 Brain Research Institute, University of Zurich and Swiss Federal Institute of Technology (ETH), Department of Biology, 8057 Zurich, Switzerland; 2 Institute of Molecular Life Sciences, University of Zurich, Winterthurerstrasse 190, CH-8057 Zurich, Switzerland

## Abstract

**Background:**

Long-distance axonal growth relies on the precise interplay of guidance cues and cell adhesion molecules. While guidance cues provide positional and directional information for the advancing growth cone, cell adhesion molecules are essential in enabling axonal advancement. Such a dependence on adhesion as well as guidance molecules can be well observed in dorsal commissural interneurons, which follow a highly stereotypical growth and guidance pattern. The mechanisms and molecules involved in the attraction and outgrowth towards the ventral midline, the axon crossing towards the contralateral side, the rostral turning after midline crossing as well as the guidance along the longitudinal axis have been intensely studied. However, little is known about molecules that provide the basis for commissural axon growth along the anterior-posterior axis.

**Results:**

MDGA2, a recently discovered cell adhesion molecule of the IgCAM superfamily, is highly expressed in dorsolaterally located (dI1) spinal interneurons. Functional studies inactivating MDGA2 by RNA interference (RNAi) or function-blocking antibodies demonstrate that either treatment results in a lack of commissural axon growth along the longitudinal axis. Moreover, results from RNAi experiments targeting the contralateral side together with binding studies suggest that homophilic MDGA2 interactions between ipsilaterally projecting axons and post-crossing commissural axons may be the basis of axonal growth along the longitudinal axis.

**Conclusions:**

Directed axonal growth of dorsal commissural interneurons requires an elaborate mixture of instructive (guidance) and permissive (outgrowth supporting) molecules. While Wnt and Sonic hedgehog (Shh) signalling pathways have been shown to specify the growth direction of post-crossing commissural axons, our study now provides evidence that homophilic MDGA2 interactions are essential for axonal extension along the longitudinal axis. Interestingly, so far each part of the complex axonal trajectory of commissural axons uses its own set of guidance and growth-promoting molecules, possibly explaining why such a high number of molecules influencing the growth pattern of commissural interneurons has been identified.

## Background

For its function the mammalian central nervous system depends on precisely organized neuronal circuits. Synaptic connections between the cells of a circuit are established during development when axonal growth cones grow along specific pathways, reaching even very distant targets with exceptionally high precision. A combination of cell adhesion molecules, surface receptors and axon guidance molecules enables the growth cone to invade permissive areas and grow along specific molecular gradients [1,2]. Long distances are covered by splitting the entire trajectory into smaller segments with intermediate targets [3]. Such intermediate targets, also called choice points, mark the end of one segment and the beginning of another. At choice points the growth cone morphology as well as the axonal trajectory change dramatically, often leading to temporary stalling and a decrease in growth rate [4]. Choice points have first been described in invertebrates such as grasshopper or *Drosophila*, where these intermediate targets are represented by specific cells called guidepost cells, whose ablation leads to axon stalling and miss-projections [3].

One of the best studied choice points in vertebrates is the ventral midline, where specialized cells called floor-plate cells selectively regulate axon crossing in bilaterally symmetric animals [2,5]. While some axons are attracted by the floor plate, others are selectively repelled. Cell populations whose axons are attracted by the floor plate are dorsolateral commissural interneurons (dI1 and dI2) [2]. Upon reaching the midline, commissural axons cross the floor plate to reach the contralateral side, where they turn orthogonally into the longitudinal axis, growing either along the floor-plate or extending laterally to join the ventral or lateral funiculus [2,6,7].

The role of the floor-plate as an important choice point for commissural axons has been clearly demonstrated in several studies [8-10]. The floor-plate-derived molecule netrin-1 was identified as the major chemoattractant for dorsolaterally located commissural axons [8]. Inactivation of either netrin-1 or its receptor, DCC (Deleted in colorectal cancer), causes severe miss-projections of commissural axons, leaving only few axons reaching the midline correctly [8,9]. No axons reached the midline when *netrin-1^-/- ^*mice were treated with the Shh inhibitor cyclopamine, demonstrating a role of Shh not only as a morphogen but also as a guidance molecule that cooperates with the chemoattractant netrin-1 [10].

While both netrin-1 and Shh are responsible for attracting commissural axons towards the ventral midline, other short-range guidance cues and adhesion molecules govern midline crossing. The best-studied molecules in this context are cell adhesion molecules of the immunoglobulin superfamily, such as axonin-1/TAG-1, NgCAM/L1, NrCAM, nectins and SynCAMs/Nectin-like molecules (Necls) [11-13]. Axonin-1 is highly expressed by commissural interneurons, whereas NrCAM, Nectin3, and SynCAM2/Necl3 are strongly up-regulated in floor-plate cells during the period of axonal midline crossing. Direct evidence for a role of these molecules in commissural axon outgrowth came from *in vivo *perturbation assays demonstrating that, in the absence of axonin-1/NrCAM, heterophilic nectin or heterophilic SynCAM interactions, axons either failed to enter and cross the floor plate or had problems turning into the longitudinal axis [11-13].

Aberrant pathfinding at the ventral midline was also found when ephrinB/EphB signalling was perturbed [14,15] or in the absence of F-spondin function [16]. While F-spondin seemed to regulate the turning angle of commissural axons, the morphogens Shh and Wnt were shown to be required for post-crossing commissural axon guidance [17-19]. In the mouse, *Wnt4 *is expressed in a decreasing anterior-to-posterior gradient in the floor plate and attracts post-crossing commissural axons rostrally in a Frizzled3-dependent manner [17]. *Shh *was found to be expressed in an opposite gradient, with the highest expression levels in the caudal spinal cord. *In vivo *loss- and gain-of-function studies demonstrated that Shh was required for the correct navigation of post-crossing commissural axons in the chicken spinal cord and that this effect was mediated by hedgehog-interacting protein (Hhip) [18]. Intriguingly, in chicken, *Wnts *were not expressed in a gradient along the anteroposterior axis. Rather, a Wnt activity gradient was formed by the graded expression of the Wnt antagonist *Sfrp1 *(*secreted frizzled related protein 1*), which was shaped by Shh [19]. Both a *Wnt *activity gradient and a *Shh *expression gradient provide directional information for post-crossing commissural axons. However, it is less clear whether they also affect neurite elongation directly. Interestingly, Avraham and colleagues [20] have recently shown that the axons of contralaterally and ipsilaterally projecting dI1 interneurons intermingle during longitudinal growth, suggesting that adhesive interaction between different fascicles might be a driving force for longitudinal growth.

Recently, we have isolated two novel cell adhesion molecules of the immunoglobulin superfamily, called MDGA1 and MDGA2, which are exclusively expressed in the peripheral and central nervous system [21]. Rat MDGA1 shows high expression levels in developing dI1 interneurons, whereas rat MDGA2 is predominantly expressed in spinal motoneurons and some subpopulations of spinal interneurons [21]. In chicken, MDGA1 has been shown to be highly expressed in motoneurons and in the floor plate [22]. In contrast to the rat counterpart, expression of chicken MDGA1 in commissural interneurons is rather low and can be observed only at later stages, raising the question of whether some functions of the mammalian MDGA1 are covered by the MDGA2 homolog in chicken. In order to study MDGA2 function *in vivo*, we have cloned the chicken *MDGA2 *ortholog. Chicken *MDGA2 *RNA is highly expressed in commissural interneurons and dorsal root ganglia (DRG) neurons. Using RNA interference (RNAi) and function-blocking antibodies, we were able to show that MDGA2 plays a crucial role in the growth of commissural axons along the longitudinal axis, suggesting that MDGA2 is required for axonal elongation, whereas Wnt and Shh signalling controls the growth direction of commissural axons after midline crossing.

## Results

With the development of *in ovo *RNAi as a tool for specific gene silencing, the chicken embryo became a powerful system to study gene function during development [23]. We thus decided to use the chicken embryo for studies addressing MDGA function. Database searches indicated that the chicken genome, as observed for other vertebrate species, contains two *MDGA *genes. The domain organization of the chicken MDGA proteins is identical to that of the corresponding rat ortholog: six immunoglobulin C-2 type repeats are combined with a fibronectin type III repeat and a MAM domain. Amplification of *MDGA2 *resulted in the identification of two different cDNA transcripts. One transcript shows an identical organization to rat *MDGA2*, and the other transcript has an insertion of 48 bp at position 2,032. Conservation between chicken and rat MDGAs is in the range 80% to 90%, suggesting that rat and chicken proteins might have conserved functions. For more details on the conservation and the phylogenetic relation of MDGAs, see Additional files 1 and 2.

### *MDGA2 *transcripts are highly expressed in spinal interneurons

**Figure 1:**
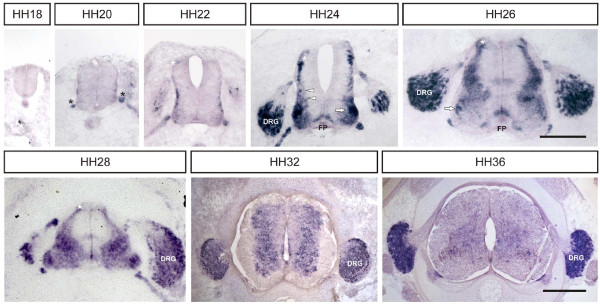
**Expression of *MDGA2 *mRNA in the chicken spinal cord**. Cross-sections of lumbosacral chicken spinal cords were incubated with Dig-labelled *MDGA2 *antisense RNA and the resulting RNA complexes were visualized using AP-conjugated anti-Dig antibodies. At stage 20, expression is mainly restricted to boundary cap cells at the ventral motor exit points (black asterisks). At stages 24 and 26, *MDGA2 *mRNA is clearly detectable in commissural interneurons (white asterisks). Starting at stage 24, transcripts can also be observed in dI6/V0 (white arrowheads) and ventrally located V1 interneurons (white arrow) and in a subpopulation of DRG neurons. Between stages 24 and 26 a weak transient expression of *MDGA2 *transcripts can also be seen in floor-plate cells (FP) and in various interneuron subpopulations. By stage 24, expression of *MDGA2 *transcripts become up-regulated in different motoneuron subpopulations. Expression levels of *MDGA2 *transcripts in the spinal cord gradually decrease after stage 28, and by stage 36 only expression in DRG neurons and a subpool of motoneurons persists. The embryonic (Hamburger-Hamilton (HH) stages are indicated. Scale bars represent 200 μm for stages 18 to 26 and 400 μm for stages 28, 32 and 36.

As *MDGA1 *expression in the spinal cord during the period of commissural axon outgrowth has recently been described [22], we decided to focus our attention on the expression of *MDGA2*. In contrast to chicken *MDGA1*, which is not or only weakly expressed by dorsally located dI1 commissural interneurons, *MDGA2 *transcripts could be readily detected in this neuronal subpopulation (white asterisk in Figure 1, stage 22; for information on chicken stages see [24]; for details on the identity of commissural interneurons see Additional file 3). Expression started as early as stage 22, when dorsal commissural axons had just entered the floor plate. Expression was up-regulated by stage 24, the age at which commissural axons made a sharp turn into the longitudinal axis. Expression levels in commissural interneurons remained high until stage 28 but dropped significantly by stage 30. In addition to the expression observed in dI1 interneurons, *MDGA2 *transcripts could also be observed in other interneuron subpopulations, such as dI3 and more ventrally located V1 interneurons (arrows in Figure 1), motoneurons as well as DRG neurons. At stage 36, *MDGA2 *expression in spinal interneurons was no longer detected, but could still be seen in some motoneurons (arrowheads in Figure 1), boundary cap cells (arrows in Figure 1) and very strongly in DRG neurons.

### MDGA2 is present on growth cones and neurites of commissural and dorsal root ganglia neurons

**Figure 2:**
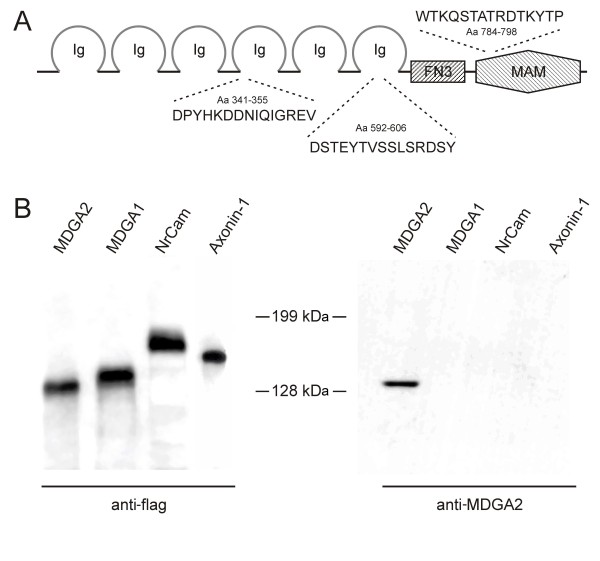
**Peptide antibodies specifically recognize the MDGA2 protein**. **(A) **Structural representation of the MDGA2 protein, with the different domains and amino acids chosen for immunization indicated. Immunoglobulin (Ig) domains are represented as open circles, the fibronectin type III repeat (FN3) is depicted as a box and the MAM domain is shown as a hexagonal structure. Two of the peptides used for immunization are located within Ig domains, the third peptide was generated against an amino acid sequence deriving from the MAM domain. **(B) **Demonstration of MDGA2 antibody specificity. Recombinant flag-tagged MDGA2, MDGA1, NrCAM and axonin-1 were separated by SDS-PAGE and the different proteins were detected by western blotting using either a monoclonal antibody against the flag tag or the newly generated MDGA2 peptide antibodies. While the flag antibody clearly recognizes all recombinant IgCAMs, the pooled MDGA2 peptide antibodies specifically recognize the MDGA2 protein. Note that even when loading higher protein concentrations or using much longer exposure times, no cross-reactivity of the MDGA2 peptide antibodies with other IgCAMs could be detected.

As a tool for our analysis, we raised peptide antibodies against different MDGA2 epitopes (Figure 2A). Two of the three peptides were designed in regions of immunoglobulin folds and one peptide was located within the MAM domain. The specificity of the antibodies was tested on a variety of flag-tagged IgCAM ectodomain fusion proteins, including MDGA2, MDGA1, NrCAM and axonin-1. Conditioned media derived from transfected 293T cells contained the expected recombinant tagged proteins as shown by western blotting with flag-specific antibodies (Figure 2B). The MDGA2 peptide antibodies only gave a signal in the lane containing MDGA2, indicating that the antisera did not cross-react with the tested IgCAM superfamily members (Figure 2B). When a full-length MDGA2 amino-terminal flag tagged construct was transfected into COS7 cells, a specific surface staining could be detected, suggesting that MDGA2 was indeed present on the surface of transfected cells, either as a glycophosphatidylinositol (GPI)-linked molecule or as a transmembrane variant (for details on surface staining and GPI anchor prediction see Additional file 4).

**Figure 3:**
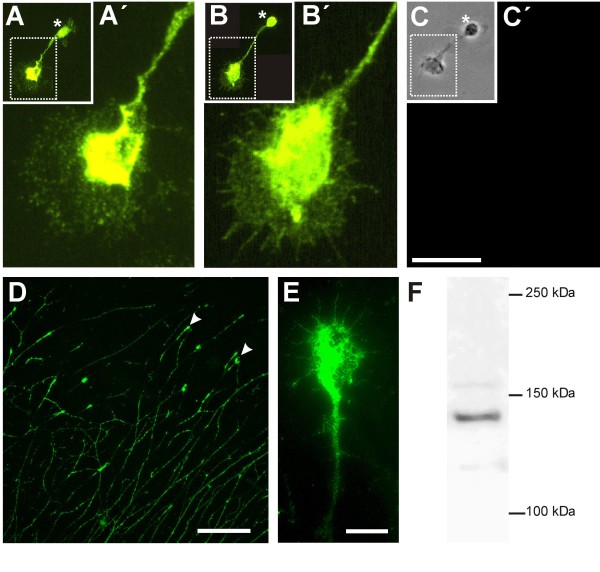
**MDGA2 is expressed on the surface of commissural interneurons and dorsal root ganglia neurons**. **(A-C) **Immunolabelling of dissociated dorsal commissural axons. Cultured dorsal commissural neurons express MDGA2 (A,A') as well as the dorsal commissural marker axonin-1 (B,B') on their surface. The position of the cell body is indicated by the asterisk. The growth cone as well as the axonal shaft (A',B') are strongly labelled in both cases whereas a control staining with the secondary antibody only (C') did not yield any labelling. (A',B') Higher magnification images of the axon shaft and growth cone of the neuron seen in (A,B), whereas (C) shows a phase contrast image of the neuron stained with the secondary antibody only (C'). **(D,E) **Immunolabelling of sensory neurons in culture. While immunostaining using MDGA2 peptide antibodies results in bright labelling of sensory growth cones (D, arrowheads), neuronal shafts are less intensely labelled. (E) Higher magnification image of a sensory growth cone. Note that not only the central part of the growth cone is stained with MDGA2 antibodies but also the extending filopodia. Scale bars: 10 μm in (A-C,E); 100 μm in (D). **(F) **Western blot analysis of DRG extracts using MDGA2 peptide antibodies. A band of around 135 kDa can be seen in protein extracts from stage 30 DRGs separated by SDS-PAGE and visualized using MDGA2 peptide antibodies.

Western blot analysis of protein extracts isolated from stage 30 DRG identified a single band with a molecular weight of 135 kDa, demonstrating that our peptide antibodies also recognized native MDGA2 protein (Figure 3F). MDGA2 peptide antibodies stained neurites as well as growth cones of dissociated dorsal commissural interneurons (Figure 3A) as well as DRG neurons (Figure 3D,E). A similar pattern was found with the dorsal commissural neuron marker axonin-1 (Figure 3B).

### Down-regulation of MDGA2 causes pathfinding errors of commissural axons

**Figure 4:**
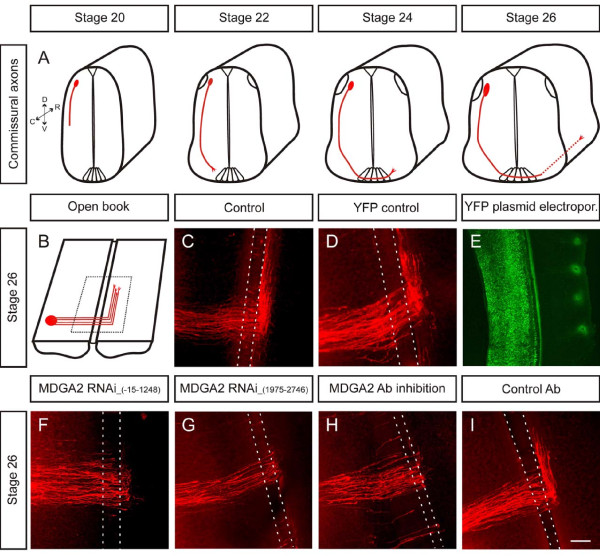
**Down-regulation of *MDGA2 *causes pathfinding errors of commissural axons**. **(A) **Schematic diagram of the time course of commissural axon pathfinding. Note that midline crossing occurs between stages 22 and 24, and rostral axon turning is initiated around stage 24. **(B) **Schematic drawing of a stage 26 open-book preparation. The red circle represents the DiI injection site labelling cell bodies of dorsolateral commissural neurons and their axons. **(C,D) **Confocal images of DiI-injected open-book preparations of an untreated control embryo (C) and an embryo injected and electroporated with a YFP control plasmid (D). Note that in both cases commissural axons make a sharp turn upon leaving the midline area and grow rostrally along the longitudinal axis of the spinal cord. The area between the dashed lines indicates the location of the floor-plate. **(E) **Electroporation efficiency with YFP. An embryo electroporated with a YFP-plasmid was imaged with epifluorescence. **(F,G) **RNAi knockdown experiments in which embryos were co-injected with long dsRNA and a YFP expression plasmid. Two independent, non-overlapping fragments were used to produce long dsRNA. Numbers in parentheses indicate the cDNA sequences used to produce dsRNA. Ipsilateral electroporation resulted in a slightly reduced number of axons reaching the contralateral side and a lack of growth of commissural axons along the longitudinal axis. Identical defects were seen with both dsRNA fragments. **(H,I) **A similar phenotype was observed when MDGA2 peptide antibodies (Ab) were injected into the central canal of stage 20 chicken embryos (H), whereas normal axon growth was seen in embryos injected with a control IgG (I). Scale bars: 100 μm.

**Figure 5:**
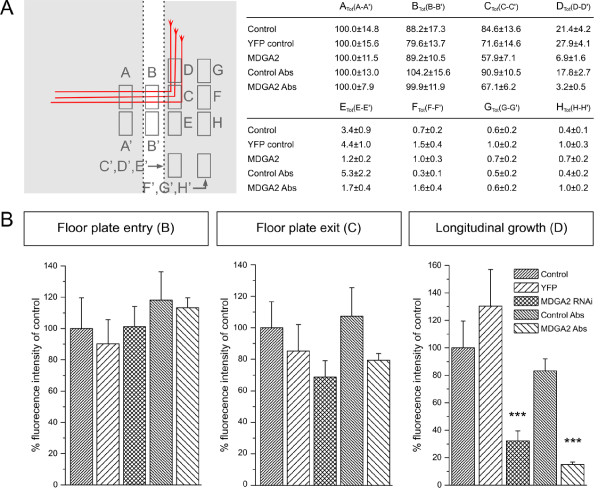
**Quantification of commissural axon outgrowth defects after perturbation of MDGA2 function by RNAi and antibody injection**. **(A) **Schematic representation of the areas used to measure fluorescence intensities in control, RNAi and antibody (Ab)-treated embryos. A, ipsilateral; B, floor plate; C, floor-plate exit; D, rostral longitudinal axis; E, caudal turn; F, no turn; G, 45° turn; H, -45° turn. Background fluorescence (A', B', C'...) at corresponding locations were subtracted from the values obtained at the different measurement sites (A, B, C....). The table shows the normalized intensities, with A_tot _(A - A') of the control situation set as the reference value (100; for details see Materials and methods). Note that the fluorescent intensities (at sites B, C, D...) for all experimental conditions are adjusted by the same factor as calculated by the normalization at the site A_tot_. **(B) **Data obtained by measurements of fluorescence intensity are presented as histograms to indicate the percentages of fluorescence intensity at particular locations. Values for control embryos were set to 100% at each of the analyzed sites. While in all analyzed conditions commissural axons were able to enter the floor plate (B, left panel), in *MDGA2 *RNAi-treated, as well as in embryos injected with the MDGA2 antibody fewer axons exited at the contralateral site (B, middle panel). The strongest difference between control and experimental animals was seen in commissural axon growth along the longitudinal axis, with only 15 to 30% of the axons extending rostrally after perturbation of MDGA2 function (B, right panel). While the effect on commissural axon stalling in the floor plate was not statistically significant, the lack of commissural axon growth along the longitudinal axis was highly significant (***P < 0.005; standard *t*-test). Error bars are given as standard errors (SEM).

To test whether MDGA2 is indeed involved in axonal growth and/or guidance, we performed RNAi-mediated knockdown experiments in commissural interneurons (Figure 4). In open-book preparations of stage 26 embryos commissural interneurons were labelled with DiI. Trajectories of dorsolateral commissural axons in control-injected embryos expressing yellow fluorescent protein (YFP) were indistinguishable from non-injected controls (Figure 4C, D). The YFP expression pattern demonstrated that the electroporation efficiency was very high, as brightly labelled YFP-positive cells could be easily detected in the electroporated half of the spinal cord (Figure 4E). In contrast to non-injected and YFP plasmid-injected embryos, embryos injected and electroporated with double-stranded RNA (dsRNA) derived from different regions of the *MDGA2 *message showed aberrant commissural axon growth (Figure 4F,G). No such change in commissural axon outgrowth was observed when dsRNA for *MDGA1 *was electroporated into the dorsolateral part of the spinal cord (Additional file 5). Electroporation of *MDGA2 *dsRNA resulted in downregulation of its mRNA on the targeted side to 28.9 ± 8.0% when compared to the unelectroporated side (for details see Material and methods). While the circumferential growth towards, as well as their entry into, the floor plate seemed to be unaffected by *MDGA2 *knockdown, both floor-plate exit and especially growth along the anteroposterior axis were clearly disturbed (Figures 4 and 5). Interestingly, this phenotype was quite different from the ones observed when axonin-1, NgCAM or NrCAM were downregulated by dsRNA ([23] and data not shown). In embryos treated with dsRNA derived from *MDGA2 *about 25% of the commissural axons were unable to reach the contralateral side and stalled within the floor plate. More importantly, the axons that made it to the contralateral side displayed no growth along the longitudinal axis (Figures 4 and 5).

The phenotype observed after *MDGA2 *knockdown could be phenocopied when MDGA2 antibodies were injected into the central canal (Figure 4H). As observed for the RNAi experiments, injections of MDGA2 antibodies did not affect the circumferential growth of commissural axons nor did they prevent them from crossing the midline (Figures 4 and 5). However, as seen after RNAi-mediated knockdown of MDGA2, post-crossing commissural axons of embryos treated with MDGA2 antibodies did not turn into the longitudinal axis. The majority of the axons stalled at the floor-plate exit site. In contrast to embryos treated with MDGA2 antibodies, embryos injected with IgG control antibodies did not show any perturbation of commissural axon growth, demonstrating that high concentrations of IgGs diffusing from the central canal did not influence axonal growth and pathfinding of commissural neurons unspecifically (Figure 4I).

### MDGA2 interacts homophilically

**Figure 6:**
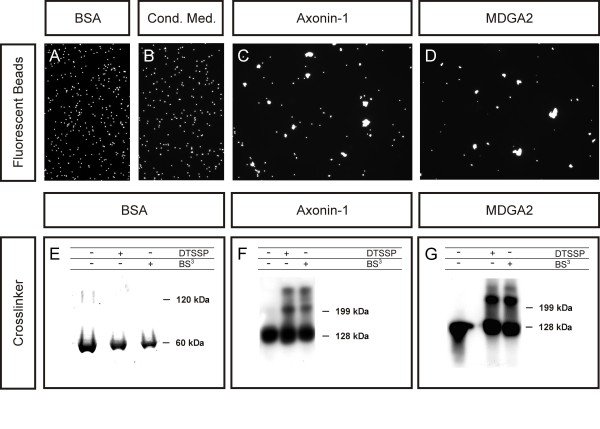
**MDGA2 is capable of interacting homophilically**. Two independent assays were used to analyze the binding capabilities of MDGA2. In an aggregation assay, fluorescent beads were coupled with BSA, proteins from conditioned medium of non-transfected cells, recombinant axonin-1 or recombinant MDGA2 and analyzed for their aggregation behaviour. **(A-D) **While no aggregates were observed with BSA (A) or beads coated with proteins released by mock-transfected cells (B), strong aggregation was detected with axonin-1- (C) and MDGA2-coupled beads (D). **(E-G) **Similar results were seen when chemical cross-linkers were used to monitor the binding capabilities of these molecules. (E) Silver staining of gels indicated that, in a concentrated BSA solution, no high molecular weight aggregates were formed in the presence of the chemical cross-linkers. Crosslinking of conditioned media containing recombinant flag-tagged axonin-1 (F) or flag-tagged MDGA2 (G) resulted in a molecular weight shift of a substantial part of the detected protein as observed by western blot assays using anti-flag antibodies. In the case of axonin-1, note that two additional bands are observed in the presence of chemical cross-linkers, indicating that axonin-1 is capable of forming multimeric complexes.

The fact that both RNAi-mediated protein knockdown as well as antibody perturbation prevented post-crossing commissural axon growth along the longitudinal axis suggested that MDGA2-mediated interactions were required for axonal elongation. As IgCAMs often engage in homophilic interactions [25], we first tested the self-aggregating activity of MDGA2 in two independent assays. Because IgCAM interactions are often of low affinity [26], we used a fluorescent bead aggregation assay as well as chemical cross-linking to visualize interactions. To test for the efficiency and the specificity of our methods, we performed a variety of control experiments, testing the binding properties of proteins known to exist as monomeric entities as well as IgCAMs known to form dimers or multimers. While bovine serum albumin (BSA)-coated beads as well as beads coated with proteins from conditioned media of control transfected HEK293T cells did not form any aggregates (Figure 6A,B), beads coated with axonin-1 formed large aggregates (Figure 6C). Beads coated with MDGA2 also resulted in the formation of large aggregates, indicating that MDGA2 was also capable of mediating homophilic interactions (Figure 6D), similar to findings for axonin-1. To independently verify the results seen in the bead aggregation assays, we used bivalent chemical cross-linkers. While the addition of chemical cross-linkers did not lead to the formation of higher molecular weight aggregates in concentrated BSA suspension (Figure 6E), axonin-1- as well as MDGA2-containing suspensions did show protein aggregates in the presence of chemical cross-linkers, indicating that these proteins can self aggregate (Figure 6F,G). As neither of our aggregation assays could discriminate between interactions occurring in *cis *or in *trans*, we additionally performed cell aggregation assays using cells transfected with full-length MDGA2. While cell suspensions of cells transfected with GFP revealed mainly single cells, transfection of cells with MDGA2 resulted in the formation of larger cellular aggregates (Additional file 6), demonstrating that MDGA2 interactions can indeed occur in *trans*.

### Ipsilaterally and contralaterally projecting axonal tracts intermingle in the longitudinal axis

**Figure 7:**
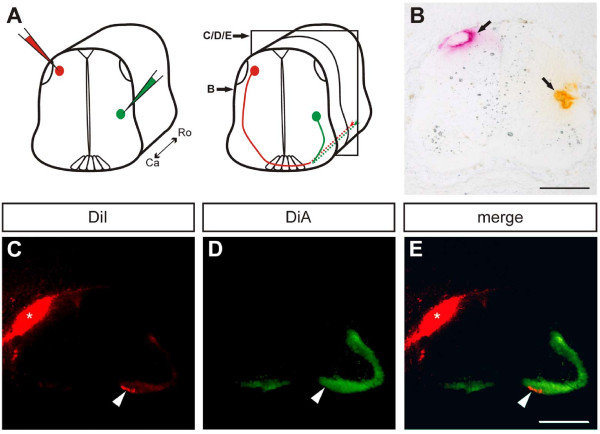
**Ipsilaterally and contralaterally projecting tracts co-localise in the ventral funiculus**. **(A,B) **The lipophilic dyes DiI (red) and DiA (green) were injected into contra- and ipsilaterally projecting neurons, respectively. Subsequently, the tissue was cut into 25-μm thick sections. Panel (B) represents the plane of dye injection, and the section shown in (C-E) was 100 μm more rostral relative to (B) (schematic drawing in (A) labels the sections). **(C) **Post-crossing axons of DiI-labelled dorsal interneurons project in the contralateral ventral funiculus (arrowhead). **(D) **DiA-labelled ipsilaterally projecting axons are found throughout the ventral funiculus, including the medial part close to the floor plate, where dorsal interneurons turn into the longitudinal axis (arrowhead). **(E) **An overlay of contra- and ipsilaterally projecting axons shows that these populations co-localize in the ventral funiculus (arrowhead). Note that due to the juxtaposition of the dorsal interneurons and the dorsal funiculus, injection of DiI also stained the longitudinal axonal tracts of the dorsal funiculus (asterisk in (B,C,E)). Ca, caudal; Ro, rostral. Scale bar: 200 μm.

As MDGA2 was found to be expressed in both contralaterally as well as ipsilaterally (dI1, V1) projecting interneurons, we tested whether MDGA2-mediated homophilic interactions would be required for axon growth along the longitudinal axis. To this end we first needed to analyze whether V1 ipsilaterally projecting and dI1 contralaterally projecting interneurons indeed intermingled in the longitudinal axis. Labelling dorsal and ventral populations of interneurons with different dyes (Figure 7A,B) resulted in the staining of contra- (DiI, Figure 7C) as well as ipsilaterally located axonal tracts (DiA, Figure 7D). Double labelling clearly indicated that ipsilateral and contralateral tracts were indeed overlapping (Figure 7E).

### Contralateral knockdown of MDGA2 causes similar turning phenotypes

**Figure 8:**
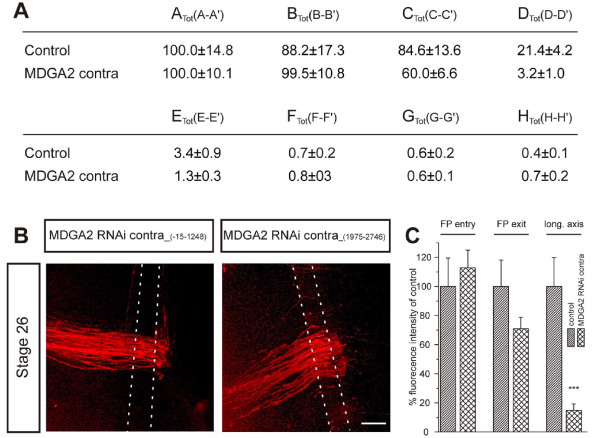
**Down-regulation of MDGA2 on the contralateral side causes phenotypes similar to those seen in ipsilateral knockdowns**. Embryos were injected with either of the two non-overlapping long dsRNA fragments covering the sequences -15 to 1,248 and 1,975 to 2,746, respectively. Following contralateral electroporation, embryos treated with either fragment did show severe defects in commissural axon growth. **(A,C) **While the table (A) gives the fluorescent intensities measured at different locations in control and RNAi-treated embryos (for details see Figure 4 and Materials and methods), the histogram (C) depicts the normalized fluorescent intensities in percentage of the control. In analogy to the results seen with ipsilateral electroporations of *MDGA2 *dsRNA (Figure 4), rostral turning of commissural axons after midline crossing in contralateral RNAi knockdown embryos is strongly reduced compared to control embryos. **(B) **Representative confocal pictures of open-book preparations of embryos after *MDGA2 *knockdown are shown. Most post-crossing commissural axons stalled at the floor-plate exit site and did not grow along the longitudinal axis. FP, floor-plate. Error bars given as standard errors (SEM). Scale bar: 100 μm.

The fact that MDGA2 was able to mediate homophilic interactions and the finding that longitudinal axon tracts formed by ipsi- and contralateral projecting axons intermingled in the longitudinal axis raised the question of whether the growth of commissural axons along the longitudinal axis depended on homophilic MDGA2 trans-interactions. To test this hypothesis, we performed knockdown experiments targeting the contralateral instead of the ipsilateral side. While most commissural axons were still able to cross the floor plate in embryos with contralateral MDGA2 knockdown, the growth of commissural axons along the longitudinal axis was almost completely abolished (Figure 8A-C). Quantification of the phenotype indicated that less than 15% of the post-crossing commissural axons were able to grow along the longitudinal axis compared to control embryos (Figure 8C). The fact that down-regulation of MDGA2 on the contralateral side had such a strong effect on the growth of dorsal commissural interneurons clearly indicated that homophilic MDGA2 interactions were required for their proper elongation along the longitudinal axis.

## Discussion

Commissural interneurons located in the dorsal part of the chicken spinal cord send out axons along very specific, highly stereotypic pathways [2]. In the lumbosacral spinal cord, outgrowth of commissural axons starts as early as stage 19, and by stage 22 commissural axons have reached the ventral midline and start crossing it [5]. By stage 25, most commissural axons have turned into the longitudinal axis and extend rostrally within the ventral funiculus before deviating from the ventral midline to join more dorsally located fibre tracts. Our experiments have shown that *MDGA2 *is strongly expressed in pre- as well as post-crossing commissural axons and that RNAi-mediated knockdown and antibody perturbation causes severe pathfinding defects in commissural axons.

**Figure 9:**
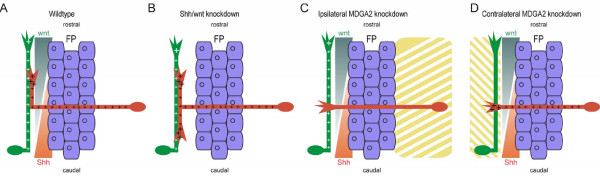
**Mechanism of commissural axon growth and guidance after midline crossing**. Schematic drawings of the commissural axon trajectory under various experimental conditions. Dorsally located commissural interneurons are depicted in red, whereas ipsilaterally projecting interneurons are shown in green. Wnt (dark green) and Shh (light red) gradients are indicated. Expression sites for *MDGA2 *are highlighted by the plus signs. Areas of *MDGA2 *knockdown are indicated by the yellow striped regions. **(A) **Under control conditions commissural axons turn rostrally to grow along the longitudinal axis towards the brain. **(B) **In embryos lacking rostrocaudal activity gradients of the guidance cues Wnt4 or Shh, commissural axons either stall or turn randomly either rostrally or caudally. **(C,D) **Ipsilateral knockdown of *MDGA2 *prevents commissural axonal growth along the longitudinal axis (C), a phenotype also seen after contralateral knockdown of *MDGA2 *(D). FP, floor-plate.

However, knockdown of MDGA2 did not influence axon outgrowth towards the floor plate, nor did it cause obvious axon stalling at the ipsilateral side. Commissural axons stalled at the floor-plate exit site and growth along the anteroposterior axis was completely abolished after *MDGA2 *knockdown or antibody perturbation. In contrast to the pathfinding defects seen after perturbation of Wnt and Shh signalling, the phenotype obtained after perturbation of MDGA2 may reflect the failure of post-crossing commissural axons to grow. In contrast to the guidance effect mediated by opposing gradients of Shh and Wnt, MDGA2-mediated commissural axon growth seems to be based on an entirely different mechanism. The fact that MDGA2 was able to form homophilic interactions and that *MDGA2 *knockdown on the contralateral side resulted in commissural axon stalling after midline crossing suggests that ipsilaterally projecting axons serve as a substrate for commissural axons to elongate (Figure 9). In accordance with this model, ventrolaterally located ipsilaterally projecting interneurons express high levels of *MDGA2 *transcripts between stages 24 and 26 (Figure 1). Therefore, MDGA2 might exert its growth-promoting effect by mediating fasciculation of post-crossing commissural axons with early established ipsilaterally projecting fibers [20,27-30]. The fact that we also see the phenotype after contralateral *MDGA2 *knockdown suggests that the observed axon outgrowth defect is not based on a failure of commissural axons to change their responsiveness to different guidance cues upon midline crossing, a mechanism recently suggested for several cases [31,32]. As neither MDGA2 in commissural neurons nor in the floor plate is targeted in the contralateral knockdown experiments, such a scenario seems unlikely. Moreover, the patterning or specification of dI1 neurons should be unaffected in contralateral *MDGA2 *knockdowns, as the unelectroporated side receives no dsRNA that could alter *MDGA2 *expression. Instead, a homophilic interaction between MDGA2 is in line with the observation that the phenotypes after ipsi- and after contralateral knockdown of *MDGA2 *are identical.

At this point it is worth mentioning that MDGA2 is also transiently expressed by floor-plate cells during the time when commissural axons cross the ventral midline. This most likely explains how perturbation of MDGA2 function could also interfere with midline crossing of commissural axons. About 25% of the commissural axons failed to cross the midline. However, due to the redundancy of IgCAMs with growth-promoting function, 75% of the axons reached the contralateral side normally. Previous studies demonstrated a growth-promoting effect of NrCAM and NgCAM [33]. Like NrCAM, MDGA1 and MDGA2 are also expressed by floor plate cells [12,34] (MG and PJ, unpublished observation). Expression of these molecules may enable commissural axons to enter and cross the ventral midline area. In this respect it will be interesting to study the binding properties of MDGA2 in more detail. While we have clearly demonstrated homophilic interactions between MDGA2 molecules, heterophilic interaction partners have not yet been identified. Putative candidates might be other members of the Ig superfamily, especially MDGA1, axonin-1, F11, NrCAM and NgCAM. Binding sites for MDGA1 within the developing spinal cord have been studied by Fujimura and colleagues [22]. Interestingly, MDGA1 binding sites only partially overlap with MDGA1 or MDGA2 expression sites, suggesting that MDGA1 does not or only weakly interacts homophilically or heterophilically with MDGA2. Using chemical cross-linkers we indeed found neither homophilic MDGA1 interactions nor did we observe the formation of MDGA1-MDGA2 heterodimers (Additional file 7).

While chicken MDGA2 is highly expressed in dorsal commissural interneurons, its rat counterpart seems to be absent or only weakly expressed in the corresponding dI1 interneurons [21]. In rat, however, MDGA1 is highly expressed in dI1 interneurons, suggesting that functions might have been shifted between rat and chicken MDGA1 and MDGA2 during evolution. Such functional shifts have already been observed for a number of proteins, such as NgCAM/L1 [35] and Wnt proteins [19]. While such species differences might initially lead to some confusion about protein function, it will ultimately help to better understand and predict functional divergence between different species, including humans.

## Conclusions

An essential mechanism enabling axonal growth is the generation of neuron-substrate interactions via cell adhesion molecules. In contralaterally and ipsilaterally projecting spinal interneurons the cell adhesion molecule MDGA2 is expressed during the period of growth along the longitudinal axis. Axons of these neuronal populations intermingle during elongation in the ventral funiculus. Elongation of dorsal commissural neurons after midline crossing is impaired when *MDGA2 *is knocked down in this neuronal population; a defect that is phenocopied when MDGA2 is downregulated on the contralateral side. Hence, for longitudinal growth of post-crossing commissural axons a homophilic interaction of MDGA2 is required.

## Materials and methods

### Cloning of chicken *MDGA2*

Reverse transcription was done on total RNA isolated from stage 26 spinal cord using either random hexamers or oligodT primers. A *MDGA2 *fragment covering the sequence between 1,966 and 2,541 was amplified using the following two degenerated primers based on the rat *MDGA-2 *sequence: *cartggacrcaratgaa *(sense) and *tgrtgrccrtacatrtg *(antisense).

The 3' end of MDGA2 was isolated using a PCR RACE (rapid amplification of cDNA ends) strategy. Using this strategy, a *MDGA2 *fragment covering the area between positions 2,226 and 3,332 could be isolated, having a designated stop codon at position 2,917. Template cDNA was generated using a specific 3' RACE primer (*cccgaattctagaagcttctcgag[T]18V*). PCR primers for the first reaction were as follows: *gaggcatatgaagtccg *(sense; 2,179 to 2,195) and *cccgaattctagaagcttc *(antisense) followed by a nested reaction with the sense primer *ggactccactattcgggt *(2,226 to 2,243).

The 5' end was cloned by PCR using the sense primer *atgttcatgttcacgtgaag**atg ***(based on the rat sequence) and specific antisense primer *ggagcactatacttgatg *(2,244 to 2,261) on a cDNA template that has been reverse transcribed from total RNA isolated from stage 26 spinal cords with a specific reverse transcription primer, *aggactgacaag*, corresponding to sequence 2,491 to 2,502. The atg sequence given in bold represents the putative atg start codon.

### *MDGA2 *expression constructs

Expression constructs for soluble (ΔGPI) and full-length MDGA2 were cloned. An EcoRI restriction site and a partial Kozak consensus sequence were added at the 5' site of the sense primer *aaaaagaattcaccatggatgtagcgatcggg*, allowing easy cloning and optimal expression. The antisense primer *aaaatgcggccgctgatcgtaaattgttggc *contains a Not restriction site at the 5' end, allowing in-frame expression. The resulting PCR fragments were cloned into the Topo pCR2 vector (TOPO^® ^TA Cloning kit; Invitrogen, Carlsbad, CA, USA) and verified by sequencing. ΔGPI fragments were subcloned in-frame into the expression vectors pcDNAI or a derivate of pMES containing either a FLAG tag (Sigma-Aldrich, St Louis, MO, USA) or a myc tag at the 3' end. Full length *MDGA2 *containing a FLAG tag introduced 3' of the signal sequence was subcloned into pcDNAI.

### *In situ *hybridization

Linearised templates spanning the *MDGA2 *sequences 1 to 1,284 and 1,285 to 2,920 were used to obtain Dig-labelled sense and antisense RNA probes (DIG RNA Labelling Kit; Roche Diagnostics GmbH, Mannheim, Germany) with T3 and T7 RNA polymerase (Roche Diagnostics).

Chicken embryos were staged as described by Hamburger and Hamilton [24]. Fixation times (4% paraformaldehyde (PFA) fixation) varied between 20 minutes (stages 14 to 20) and 1 hour (stages 36 and older). Subsequently, tissues were embedded in Tissue-Tek^® ^compound (Sakura Finetek Europe, Zoeterwoude, The Netherlands), frozen on dry ice and stored at -80°C. Sections cut at a thickness of 25 μm were collected on SuperFrost Plus slides (Menzel GmbH & Co. KG, Braunschweig, Germany) and immediately dried for 2 to 4 hours. Sections were hybridized with two RNA probes for MDGA2 (1 to 1,284; 1,285 to 2,920) using previously published protocols [36]. After hybridization, sections were developed in colouring solution (240 μg/ml levamisole, 35 μg/ml nitro blue tetrazolium, 17.5 μg/ml 5-bromo-4-chloro-3-indolyl phosphate in TBS buffer) in the dark until the desired intensity of reaction product was achieved.

### Dorsal root ganglia outgrowth

DRGs from stage 30 chicken were dissected and placed on polylysine-coated dishes and incubated at 37°C for 2 to 3 days in growth medium (DMEM/F12, 10% FCS, 0.36% methocel solution, AraC, 100 ng/ml nerve growth factor (NGF), 50 μg/ml gentamycin).

### Immunohistochemistry

DRGs were fixed in 4% PFA for 15 minutes and subsequently incubated for 30 minutes in PBS containing 2% goat serum and 0.2% fish skin gelatine. MDGA2 peptide antibodies were added for 1 h at room temperature following several wash steps with PBS and the application of the secondary antibody for 30 minutes.

### Dissociated commissural neurons

The dorsal most 25% of stage 26 spinal cords were dissected. After trypsin treatment and trituration, the dissociated neurons were cultured on polylysine-coated (10 μg/ml;Sigma) eight-well LabTek slides (Nalgene-Nunc, Thermo Fisher Scientific, Rochester, NY 14625, USA) for 24 h in growth medium (MEM with GlutaMAX-I (Invitrogen), 1 mM pyruvate, 4 mg/ml Albumax (Invitrogen), N3 (100 μg/ml transferring, 10 μg/ml insulin, 20 ng/ml triiodothyronine, 40 nM progesterone, 200 ng/ml corticosterone, 200 μM putrescine, 60 nM sodium selenite; reagents from Sigma). Cells were fixed with 4% PFA for 15 minutes, washed in PBS and blocked in PBS containing 10% FCS for 20 minutes. The cells were subsequently incubated with the primary antibodies (rabbit anti-axonin-1, rabbit anti-MDGA2) in 10% FCS/PBS for 90 minutes and washed several times with PBS. After incubation with the secondary antibody (goat anti-rabbit-Cy3; Jackson ImmunoResearch Laboratories, Inc., West Grove, PA 19390, USA), the cells were washed well in PBS and subsequently analyzed by confocal microscopy.

### Real-time PCR

Total RNA was isolated from the electroporated and the unelectroporated side of stage 26 *MDGA2 *knockdown embryos and reverse transcribed using oligodT primers. Real-time PCR was performed with two different sets of *MDGA2 *primers amplifying the regions 826 to 995 and 2,436 to 2,607 and one primer pair for an actin control (916 to 1,089) as well as a *GAPDH *control (770 to 880). Amplification of a *MDGA1 *fragment (2,439 to 2,584) on the above described cDNAs was used to demonstrate the specificity of the *MDGA2 *downregulation (*MDGA1 *levels in the *MDGA2 *dsRNA electroporated side were 91.8 ± 6.3% compared to the control non-electroporated side).

### Binding studies

Transfection of HEK293T cells was done using the PolyFect transfection reagent (Qiagen GmbH, 40724 Hilden, Germany) according to the manufacturer's instruction. Serum-free conditioned media containing recombinant MDGA2 carrying either a flag or a myc tag were concentrated to approximately 20 μg/ml using the Centricon centrifugation device (Milipore-Amicon, Billerica, MA 01821, USA). Chemical cross-linkers (bis(sulfosuccinimidyl) suberate, ethylene glycolbis(sulfosuccinimidylsuccinate), 3,3'-dithiobis(sulfosuccinimidylproprionate)) were added at a ten-fold molar excess (in respect to the present free amino groups) and incubated at room temperature for 30 minutes. The reaction was terminated by adding 10 mM Tris-buffered saline and the formed protein complexes were analyzed by western blot. Beads (Fluorosbrite PolyFluor Microspheres; Polysciences, Inc., Warrington, PA 18976, USA) were coupled according to the manufacturer's instructions with either BSA or conditioned medium containing different forms of recombinant MDGA2. Prior to the aggregation, assay beads were briefly sonicated. Aggregation was performed in a rotating shaker for exactly 30 minutes and the formed aggregates were immediately analysed by fluorescent microscopy.

### Long double-stranded RNA synthesis

Two long dsRNA fragments covering different parts of the designated cDNA sequence (-15 to 1,248 and 1,975 to 2,746) were used to down-regulate *MDGA2 *functions. Linearised plasmids containing the different fragments were transcribed with T3 or T7 polymerase according to the manufacturer's protocol (Roche Diagnostics). Following transcription, the plasmid DNA was digested with RNase-free DNase for 1 h. Single-stranded RNA molecules were then extracted once with phenol-chloroform-isoamylalcohol (25:24:1; pH 4.5) and once with chloroform-isoamylalcohol (24:1) before precipitating with ethanol. Equal amounts of sense and antisense RNA were mixed, heated for 5 minutes to 95°C and subsequently allowed to cool down to room temperature overnight. Formation of dsRNA was confirmed by non-denaturing agarose gel electrophoresis.

### *In ovo *RNAi and antibody injection

Hisex chicken embryos obtained from a local supplier were used according to regulations of the Veterinäramt des Kantons Zürich. Electroporation was done as described previously [23]. Between 0.1 and 0.5 μl dsRNA (300 ng/μl) mixed with a YFP control plasmid (20 ng/μl) and Trypan Blue (0.04% v/v; Invitrogen) were injected into the central canal of 3-day-old chick embryos (stages 18 to 20) using glass capillaries. Five pulses of 50-ms duration at 25 V were given using platinum electrodes (BTX, Genetronics, San Diego, CA, USA) of 4 mm length with a distance of 4 mm between anode and cathode. MDGA2 antibodies were raised against three different peptides whose sequences are indicated in Figure 2A. A standard immunisation protocol offered by Eurogentec was used for the antibody production (Eurogentec, 4102 Seraing, Belgium). Antibodies from the final bleed were affinity purified against the corresponding peptides. Peptide antibodies against MDGA2 (3 to 5 μg/μl) were injected into the central canal of stage 18 chicken embryos as previously described [12]. For control injections, purified IgGs from non-immunized rabbit were used at identical concentrations.

### Tracing of contra- and ipsilateral axons

Five-day-old chicken embryos were decapitated and fixed in 4% PFA/1× PBS for 1.5 h at room temperature. A 2-mm thick transverse section of the embryo (at the lumbar level of the spinal cord) was injected with lipophilic dye (FastDiI and FastDiA, 5 mg/ml in methanol; Molecular Probes, Eugene, OR, USA) at the caudal surface as indicated in Figure 7A. Sections were cryoprotected overnight with 25% sucrose in 0.1 M sodium phosphate buffer (pH 7.4), embedded in OCT Tissue-Tek and frozen on dry ice. For analysis, the tissue was cut into 25-μm thick sections on a cryostat (Leica CM1850).

### DiI injection

Open-book preparations of stage 26 chicken spinal cords were prepared as described previously [37]. Briefly, FastDiI (Molecular Probes) at a concentration of 5 mg/ml was injected into the region of dorsally located interneurons. After 2 days of incubation to allow for the diffusion of the dye, spinal cords were mounted in PBS between coverslips and analyzed by confocal microscopy. Confocal sections were taken with a step size of approximately 0.6 μm for a maximal projection of approximately 50 μm.

### Phenotype quantification

Fluorescence intensities at different locations along the trajectory of commissural axons (see schematic representation in Figure 5: A, ipsilateral; B, floor plate; C, floor-plate exit; D, rostral longitudinal axis; E, caudal turn; F, no turn; G, 45° turn; H, -45° turn) were quantified using the NIH ImageJ program [38]. Background fluorescence levels A' to H' at corresponding locations were subtracted from the values obtained at the different sites of measurement (A_tot_(A - A'), B_tot_(B - B')...). The raw data are given in the table in Figure 5A. Subsequently, fluorescence intensities were normalized to the control values, with the control representing 100% at each given location. Injection sites (n between 40 and 60) were quantified using the program Prism 4 (GraphPad Software Inc., La Jolla, CA 92037, USA) and the obtained data were converted into a histogram (Figure 5B) showing the percentage of commissural axon intensity being present at particular locations compared to control values (floor-plate, floor-plate exit, rostral turn).

## Abbreviations

bp: base pair; BSA: bovine serum albumin; DMEM: Dulbecco's modified Eagle's medium; DRG: dorsal root ganglia; dsRNA: double-stranded RNA; EST: expressed sequence tag; FCS: foetal calf serum; GFP: green fluorescent protein; GPI: glycophosphatidylinositol; Necl: Nectin-like; PBS: phosphate-buffered saline; PFA: paraformaldehyde; RNAi: RNA interference; Shh: Sonic hedgehog; YFP: yellow fluorescent protein.

## Competing interests

The authors declare that they have no competing interests.

## Authors' contributions

PJ, RB, AW, EI, IA, ES and MG carried out experiments and read and approved the final version of the manuscript. PJ, AW and MG analyzed the data and prepared the final figures. MG conceived the study and wrote the manuscript.

## Supplementary Material

Additional file 1 **Conservation and phylogenetic relation of *MDGA *transcript and protein sequences**. *MDGA *sequences used for comparisons and phylogenetic analysis were manually annotated, using combined information from EST and genome databases (GeneBank and Ensembl, version 50/51, 2008). Human and mouse sequences were used as initial query (for more details on sequences annotation see [39]). Transcript and protein sequences of MDGAs were pair-wise aligned using the blast two sequences program. **(A) **Comparison between human (hs), mouse (mm) and chicken (gg) MDGA protein sequences. The percentage of identical amino acids between species is given in bold numbers, whereas the percentage of conserved amino acids is given in parentheses. Conservation between orthologs is highlighted in green, whereas conservation between MDGA1 and MDGA2 is given in red. **(B) **Comparison between coding sequence of human (hs), mouse (mm) and chicken (gg) *MDGA*s. The percentage of identical nucleotides between the sequences is given. Conservation between orthologs is highlighted in green, whereas conservation between *MDGA1 *and *MDGA2 *is shown in red. **(C) **Phylogeny of MDGA proteins. In order to cover a broad spectrum of different species, three mammalian (hs, *Homo sapiens*; mm, *Mus musculus*; rn, *Rattus norvegicus*), one marsupial (md, *Monodelphis domestica*), two avian (tg, *Taeniopygia guttata*; gg, *Gallus gallus*), one reptilian (ac, *Anolis carolinensis*) and one amphibian (xt, *Xenopus tropicalis*) species were used. As an outgroup to root the tree, an MDGA homolog found in *Ciona intestinalis *was included. Sequences were aligned using MUSCLE [40]. A conserved stretch of 809 amino acids determined by the program Gblocks [41] was used for phylogenetic reconstruction. The phylogenetic tree was built using the maximum likelihood method with the WAG amino acid replacement matrix. The approximate likelihood ratio test (aLRT) was used to judge branch reliability. aLRT values above 0.5 are shown. Avian proteins are shown in red. The scale bar represents the percentage (0.3 equals 30%) of amino acid substitutions required to generate the corresponding tree. For more details on phylogenetic analysis and the programs used, see [39]. Click here for file

Additional file 2 **Fasta formats of MDGA proteins**. Amino acid sequences of the different MDGA proteins. Positions denoted as 'Xs' represent unknown amino acids that could not be deduced from the corresponding genome or EST database as these sequence stretches were not yet covered. Click here for file

Additional file 3 ***MDGA2 *is expressed in dI1 and additional interneuron subpopulations**. **(A) **Schematic drawing of a cross-section through the chicken spinal cord. The red square represents the section of the spinal cord depicted in B,C,E,F. **(B,C,E,F) ***In situ *hybridisations for *MDGA2 *and the commissural marker LHX2 were performed on consecutive cryosections of stage 24 (B,C) and stage 26 (E,F) chicken spinal cords. At both stages *MDGA2 *is expressed in LHX2-positive cells (arrowheads), confirming the expression of *MDGA2 *in commissural dI1 interneurons. Note that *MDGA2 *is also highly expressed in more ventrally located interneurons, which are not stained with LHX2. **(D) **At stage 26, LHX2-positive cells at the dorsal border of the spinal cord also co-localise with cells expressing the MDGA2 protein. Click here for file

Additional file 4 **MDGAs are predicted GPI-anchored proteins and recombinant chicken MDGA2 is present on the surface**. **(A) **GPI-anchor prediction. MDGA sequences were analyzed using the PredGPI predictor [42]. Sequences of chicken, human and rat MDGA proteins were used. For all six sequences, a putative GPI-anchor was predicted, with the highest probabilities for rat MDGA1, human MDGA2 and mouse MDGA2. The lowest probability was seen in chicken MDGA1. Predicted omega-site positions (position after which cleavage of the polypeptide chain occurs) are highlighted in red and the specificity of GPI anchor attachment to this site is shown in green. Similar results were obtained using the GPI-SOM program [43]. **(B) **Comparison of the putative GPI-anchoring sequences of the MDGA proteins to membrane dipeptidase (MDP). When compared with the GPI anchoring sequence of MDP [44,45], MDGAs share the anchoring characteristics. The ω-site is followed by a spacer region and a hydrophobic stretch. The different regions are indicated. **(C) **Recombinant full-length MDGA2 is targeted to the cell membrane. Live stainings on cells transfected with a full-length MDGA2 flag-tagged construct resulted in surface staining with anti-flag antibodies, whereas cells transfected with a construct lacking the putative GPI anchoring sequence showed no staining. Anti-actin antibodies did not stain living cells. In permeabilised cells anti-flag staining resulted in a typical endoplasmic reticulum/Golgi staining with either MDGA2 construct, demonstrating that these constructs are processed in the secretory pathway. Under these conditions, cytoskeletal actin staining can also be observed with anti-actin antibodies. Note that the cell density in both cultures is identical, but that only around 20% of the cells were transfected with the MDGA2 full-length or MDGA2ΔGPI plasmids. For the live staining pre-cooled cells were incubated with the primary antibody in culture medium for 30 minutes at 4°C. The antibody was washed away with culture medium and the cells were fixed in PFA for 30 minutes at 4°C. After several wash steps in PBS the cell were incubated with secondary antibody, washed again and imaged with a fluorescent microscope. For the permeabilised conditions cells were fixed and permeabilised using triton before adding the primary antibody. Click here for file

Additional file 5 **RNAi-mediated knockdown of *MDGA1 *had no effect on commissural axon pathfinding**. **(A) **Electroporation of *MDGA1 *dsRNA into the dorsolateral spinal cord did not alter the growth pattern of dI1 commissural interneurons. Numbers in parentheses indicate the cDNA sequence of *MDGA1 *used to produce dsRNA. Scale bars: 100 μm. Click here for file

Additional file 6 **Homophilic MDGA2 interactions can be mediated via *trans *aggregation**. Cell aggregation assays were performed in suspensions of COS7 cells. In these assays 3.5-cm dishes of adherent COS7 cells (60 to 70% confluence) were transfected with either *GFP *containing plasmid (0.7 μg/dish) or *MDGA2 *full-length constructs (3.5 μg/dish). After 36 h cells were treated with Trypsin/EDTA and the detached cells were re-suspended in 2 ml of cell culture medium (DMEM, 10% FCS) and incubated in a horizontally stored 50-ml Falcon tube for 45 minutes. Subsequently, cell suspensions were titurated and kept in culture under mild shaking conditions for another 10 minutes. After this period an aliquot was removed and the formation of cellular aggregates was analysed in a Neubauer chamber. **(A,B) **While in cell suspensions of cells transfected with full-length *MDGA2 *large cellular aggregates could be observed (A,A'), GFP-transfected COS7 cells were mainly single cell suspensions (B,B'). As membrane associated cell adhesion molecules are oriented at the cell surface, the formation of cellular aggregates is an indication for a *trans*-interaction of MDGA2. (B,B') Phase contrast pictures overlaid with GFP fluorescence (bright cells). Note that transfection efficiency is in the range of 70% but under the depicted condition only cells expressing high levels of GFP could be detected. Scale bar: 50 μm. Click here for file

Additional file 7 **MDGA1 and MDGA2 do not interact heterophilically**. Western blot analysis using anti myc antibodies. **(A) **The addition of cross-linkers to serum free conditioned media containing recombinant myc-tagged MDGA1ΔGPI did not lead to higher molecular weight aggregates, suggesting that MDGA1 does not interact homophilically. **(B) **The combination of myc-tagged MDGA1ΔGPI with flag-tagged MDGA2ΔGPI also did not reveal any higher molecular weight aggregates when stained with myc antibodies, indicating that MDGA1 also forms no heterophilic interactions with MDGA2. Click here for file
