# Supplementary figures and images for: Rostral growth of commissural axons requires the cell adhesion molecule MDGA2
Source: Neural Dev. 2011 May 4;6:22. doi: 10.1186/1749-8104-6-22 (PMC3113314; doi:10.1186/1749-8104-6-22)

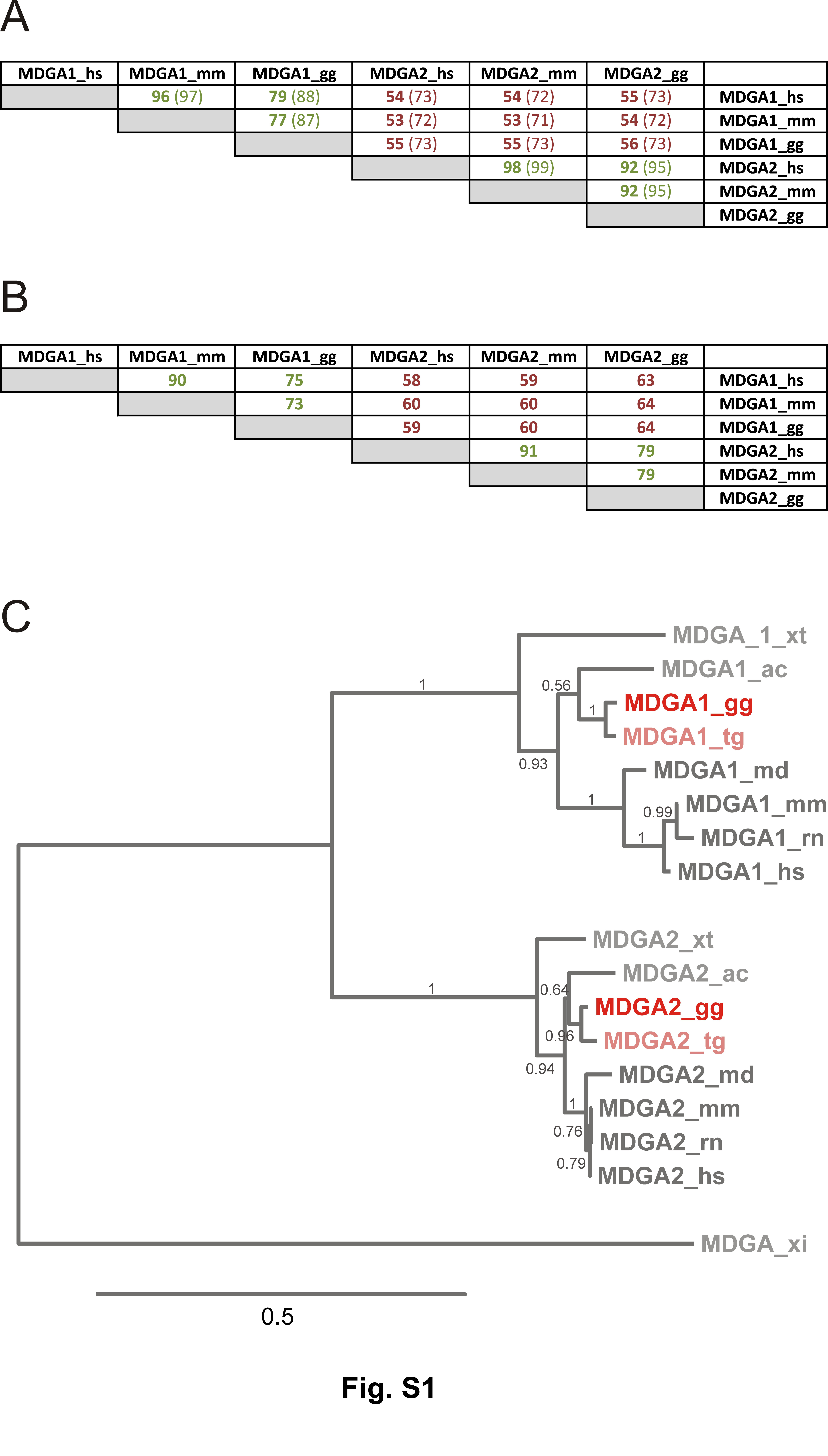

Supplement: Additional file 1 — Conservation and phylogenetic relation of MDGA transcript and protein sequences. MDGA sequences used for comparisons and phylogenetic analysis were manually annotated, using combined information from EST and genome databases (GeneBank and Ensembl, version 50/51, 2008). Human and mouse sequences were used as initial query (for more details on sequences annotation see [39]). Transcript and protein sequences of MDGAs were pair-wise aligned using the blast two sequences program. (A) Comparison between human (hs), mouse (mm) and chicken (gg) MDGA protein sequences. The percentage of identical amino acids between species is given in bold numbers, whereas the percentage of conserved amino acids is given in parentheses. Conservation between orthologs is highlighted in green, whereas conservation between MDGA1 and MDGA2 is given in red. (B) Comparison between coding sequence of human (hs), mouse (mm) and chicken (gg) MDGAs. The percentage of identical nucleotides between the sequences is given. Conservation between orthologs is highlighted in green, whereas conservation between MDGA1 and MDGA2 is shown in red. (C) Phylogeny of MDGA proteins. In order to cover a broad spectrum of different species, three mammalian (hs, Homo sapiens; mm, Mus musculus; rn, Rattus norvegicus), one marsupial (md, Monodelphis domestica), two avian (tg, Taeniopygia guttata; gg, Gallus gallus), one reptilian (ac, Anolis carolinensis) and one amphibian (xt, Xenopus tropicalis) species were used. As an outgroup to root the tree, an MDGA homolog found in Ciona intestinalis was included. Sequences were aligned using MUSCLE [40]. A conserved stretch of 809 amino acids determined by the program Gblocks [41] was used for phylogenetic reconstruction. The phylogenetic tree was built using the maximum likelihood method with the WAG amino acid replacement matrix. The approximate likelihood ratio test (aLRT) was used to judge branch reliability. aLRT values above 0.5 are shown. Avian proteins are shown in red. [file 1749-8104-6-22-S1.JPEG]

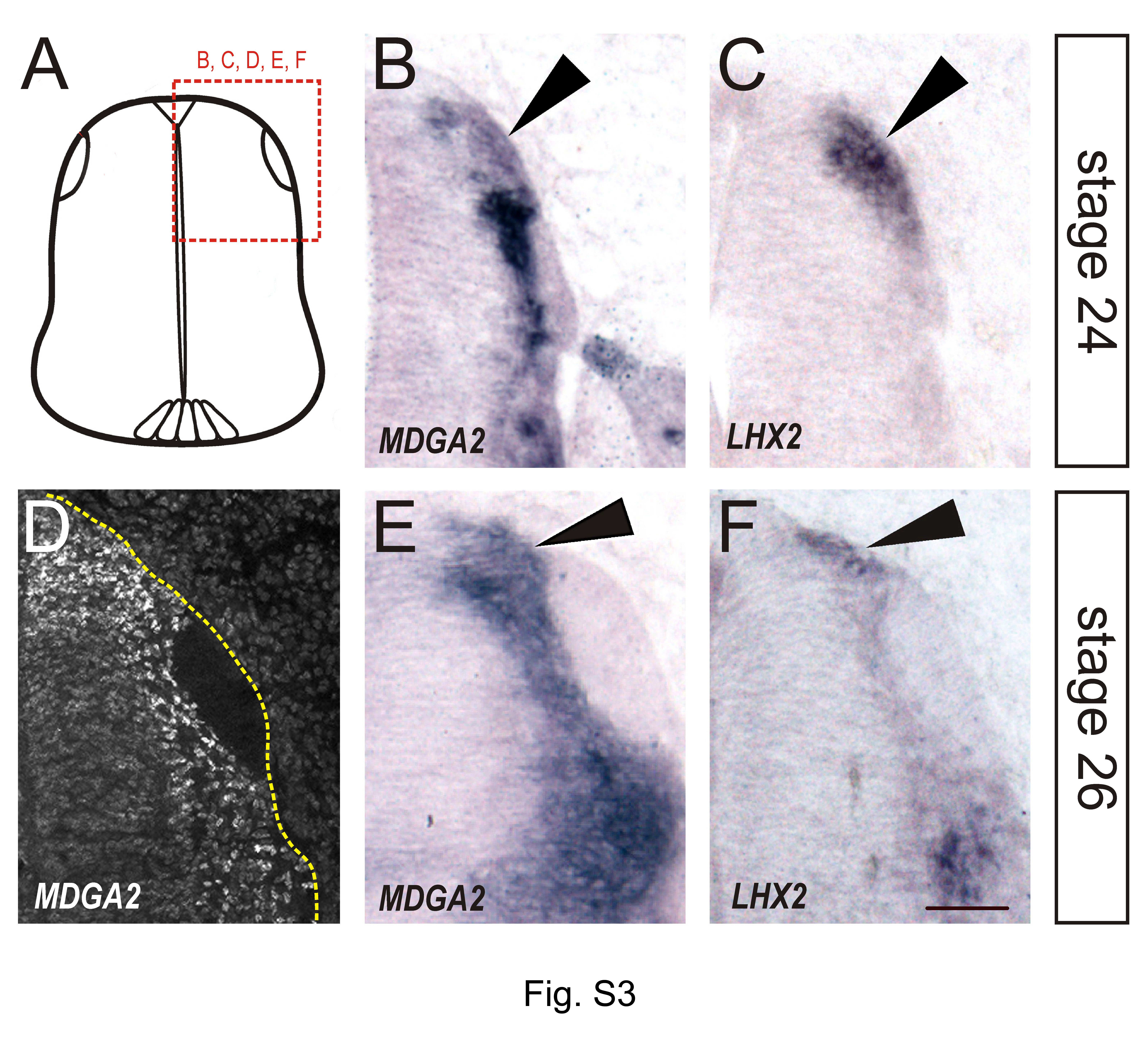

Supplement: Additional file 3 — MDGA2 is expressed in dI1 and additional interneuron subpopulations. (A) Schematic drawing of a cross-section through the chicken spinal cord. The red square represents the section of the spinal cord depicted in B,C,E,F. (B,C,E,F) In situ hybridisations for MDGA2 and the commissural marker LHX2 were performed on consecutive cryosections of stage 24 (B,C) and stage 26 (E,F) chicken spinal cords. At both stages MDGA2 is expressed in LHX2-positive cells (arrowheads), confirming the expression of MDGA2 in commissural dI1 interneurons. Note that MDGA2 is also highly expressed in more ventrally located interneurons, which are not stained with LHX2. (D) At stage 26, LHX2-positive cells at the dorsal border of the spinal cord also co-localise with cells expressing the MDGA2 protein. [file 1749-8104-6-22-S3.JPEG]

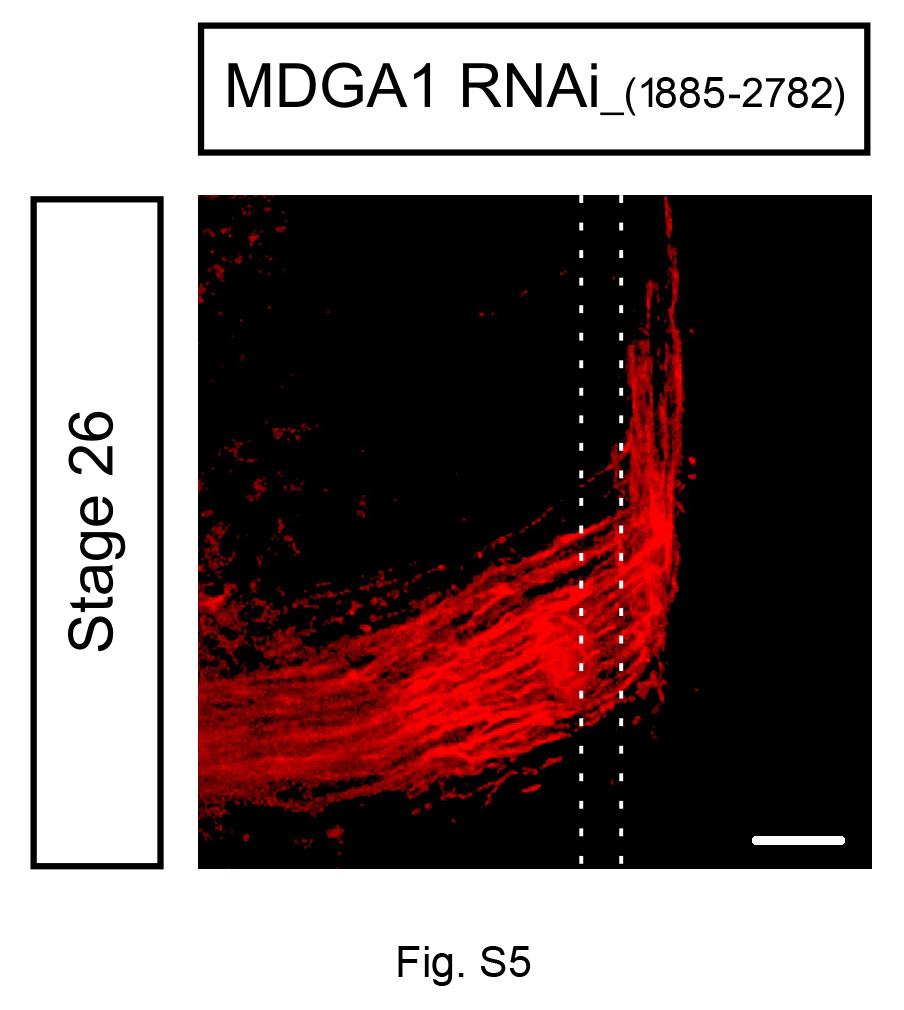

Supplement: Additional file 5 — RNAi-mediated knockdown of MDGA1 had no effect on commissural axon pathfinding. (A) Electroporation of MDGA1 dsRNA into the dorsolateral spinal cord did not alter the growth pattern of dI1 commissural interneurons. Numbers in parentheses indicate the cDNA sequence of MDGA1 used to produce dsRNA. Scale bars: 100 μm. [file 1749-8104-6-22-S5.JPEG]

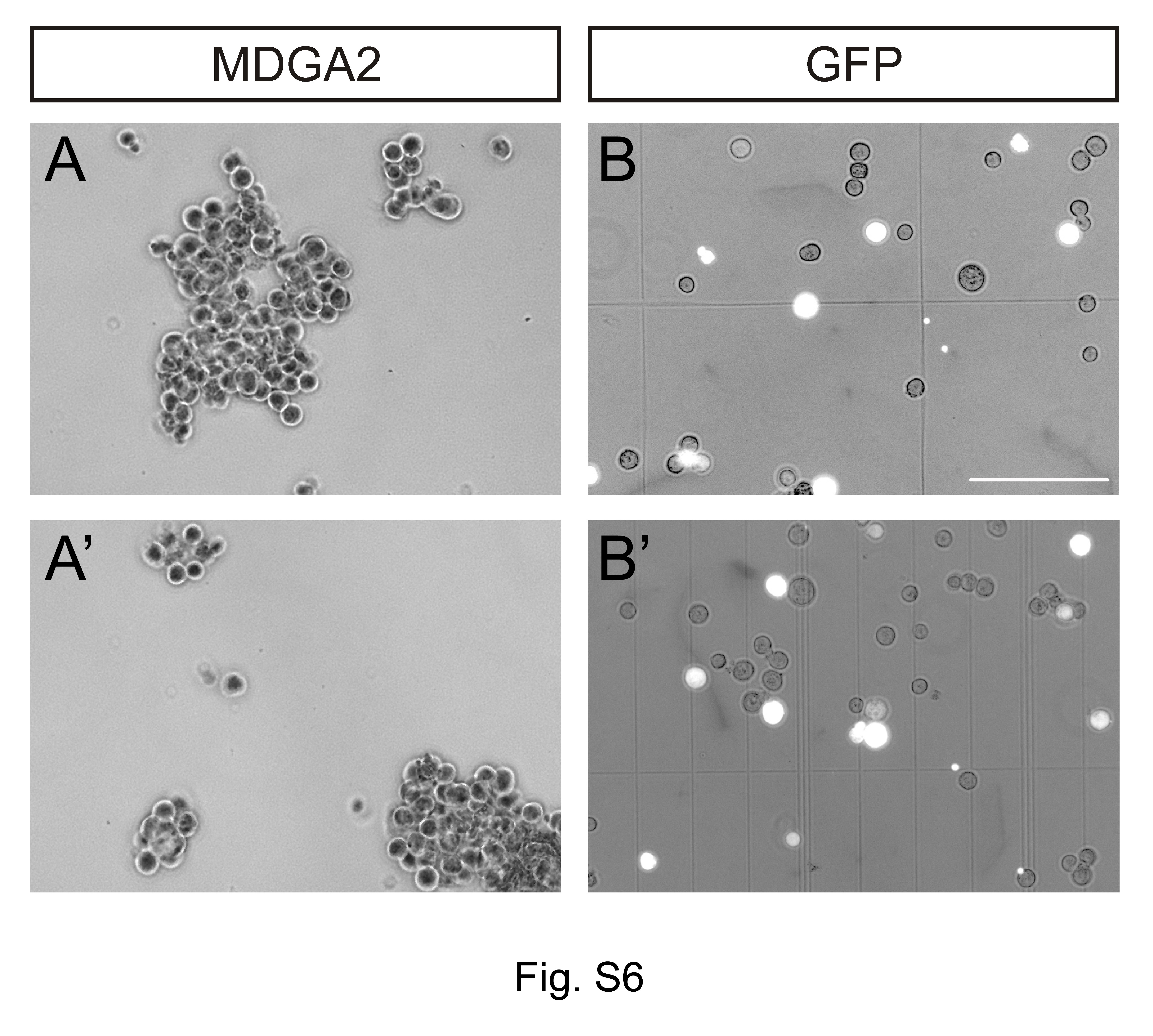

Supplement: Additional file 6 — Homophilic MDGA2 interactions can be mediated via trans aggregation. Cell aggregation assays were performed in suspensions of COS7 cells. In these assays 3.5-cm dishes of adherent COS7 cells (60 to 70% confluence) were transfected with either GFP containing plasmid (0.7 μg/dish) or MDGA2 full-length constructs (3.5 μg/dish). After 36 h cells were treated with Trypsin/EDTA and the detached cells were re-suspended in 2 ml of cell culture medium (DMEM, 10% FCS) and incubated in a horizontally stored 50-ml Falcon tube for 45 minutes. Subsequently, cell suspensions were titurated and kept in culture under mild shaking conditions for another 10 minutes. After this period an aliquot was removed and the formation of cellular aggregates was analysed in a Neubauer chamber. (A,B) While in cell suspensions of cells transfected with full-length MDGA2 large cellular aggregates could be observed (A,A'), GFP-transfected COS7 cells were mainly single cell suspensions (B,B'). As membrane associated cell adhesion molecules are oriented at the cell surface, the formation of cellular aggregates is an indication for a trans-interaction of MDGA2. (B,B') Phase contrast pictures overlaid with GFP fluorescence (bright cells). Note that transfection efficiency is in the range of 70% but under the depicted condition only cells expressing high levels of GFP could be detected. Scale bar: 50 μm. [file 1749-8104-6-22-S6.JPEG]

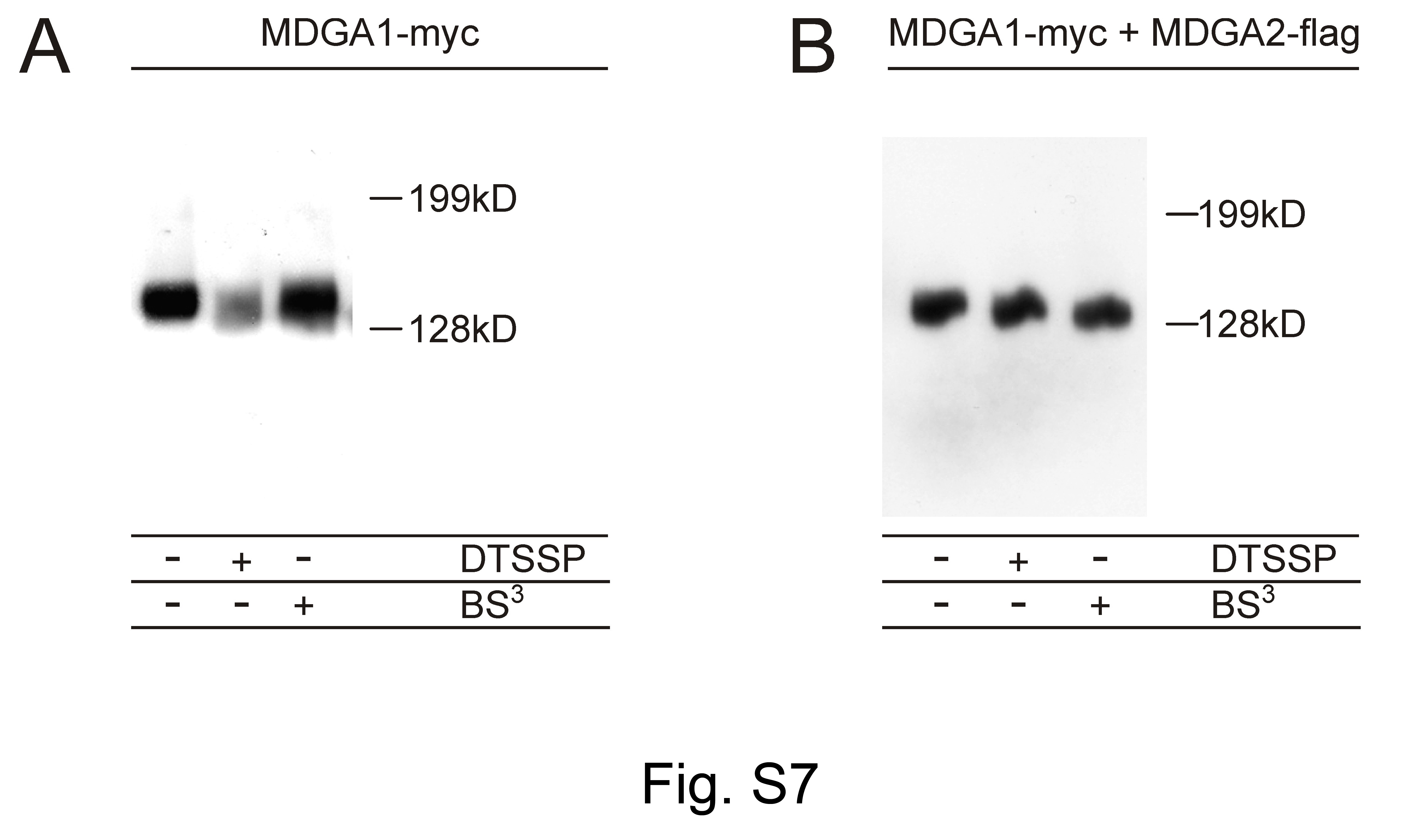

Supplement: Additional file 7 — MDGA1 and MDGA2 do not interact heterophilically. Western blot analysis using anti myc antibodies. (A) The addition of cross-linkers to serum free conditioned media containing recombinant myc-tagged MDGA1ΔGPI did not lead to higher molecular weight aggregates, suggesting that MDGA1 does not interact homophilically. (B) The combination of myc-tagged MDGA1ΔGPI with flag-tagged MDGA2ΔGPI also did not reveal any higher molecular weight aggregates when stained with myc antibodies, indicating that MDGA1 also forms no heterophilic interactions with MDGA2. [file 1749-8104-6-22-S7.JPEG]
