# Supplementary material for: Rostral growth of commissural axons requires the cell adhesion molecule MDGA2
Source: Neural Dev. 2011 May 4;6:22. doi: 10.1186/1749-8104-6-22 (PMC3113314; doi:10.1186/1749-8104-6-22)
Supplement: Additional file 2 — Fasta formats of MDGA proteins. Amino acid sequences of the different MDGA proteins. Positions denoted as 'Xs' represent unknown amino acids that could not be deduced from the corresponding genome or EST database as these sequence stretches were not yet covered. [file 1749-8104-6-22-S2.DOC]

>MDGA1_ac

METLWVLLLSLVPACAKGQGVYAPAQAQIIHAGQACVVKEDNISERVYTIREGDTLVLQCLVTGHPRPQVRWTKTAGSASDKFQETSVFNETLRIEKIQRLQGGRYYCKAENGVGVPAIRSIRVDVQYLDEPVLTVHQTISDVRGSFYQEKTVFLRCTVNSNPPARFIWRRGSETLSHSQDNGVDIYEPLYTQGETKVLKLKNLRPQDYASYTCQVSVRNVCGIPDKSITFALTNTTAPPSLKLSVNETLVINPGDNITIQCSLTGGDPLPRVIWSHSPSPMPRNSLIQGSNLTIWSIRVEDSGYYNCTAINNVGNPAKKTVNLLVRSMRNATFQITPDVIKESETIQLGQDLKLSCHVDAVPQEKVFYFWYKNGKLAKLSDRLVIIRNDPELPPVTSSLEIIDLRFTDYGTYLCVASFQGAPIPDLSVEVNISSETVPPTIMVPKGQSTVTVREGSRAELQCEVRGKPKPPIIWSRVDKEAPMPSGAMTVETSDGKLHLERVTREMSGTYKCQTARYNGFNIRSREALVQLNVQFPPVVEPVFQDIRQAVGRSVTLRCTMLKGSPMKVATAVWRFNGSLLTVPPAEQQDYSEYKVDSLSRETSGSYECSISNDVGVATCLFQVSGKAYNPEFYYDTPNPTRKQSKNYSYVLQWTQKEPDAVDPILSYRLDVRQMNQRDLPPKSISVRKMEKGMLQEHLLTDLKVPQSYEVRLTPITRFGTGDMATRMIHYLERKLEPLQGCNTCHFEDEKICGYTQDTIDNFDWIRQSSLTHDPKRSANTGPTMDFSGTPEGYYMFIEASAPRVKDDKARLISPMYNMTARFCVSFYYHMYGKHIGSLNLLVHVKNKQPTQALSIKGDQGNLWQQAHVPINPAGPFQIIFEGVRGTGSEGDIAIDDVTLKKGDCPRRPTAPNKAVALPGNGAVTHHKSFLCGPLLFFLYVLLR

>MDGA1_gg

MEMICVLFLSLVPAYSRGQGVYAPAQAQIIHAGQACVVKEDNISERVYTIREGDTLVLQCLVTGHPRPQVRWTKTAGSASDKFQETSVLNETLRIEKIQRLQGGRYYCKAENGVGVPAIKSIRVDVQYLDEPVLTVHQTISDVRGSFYQEKTVFLRCTVNSNPPARFIWKRGAETLSHSQDNGVDIYEPLYTQGETKVLKLKNLRPQDYASYTCQVSVRNVCSIPDKSITFQLTNTTAPPALKLSVNETLVVNPGDNVTMQCSLTGGDPQPEVLWSHSPGPLPPNSLVQGGNLTIWRIRVEDSGYYNCTAINNVGNPAKKTVNLPVRSMKNATFQITPDVIKESETIQLGQDLKLSCHVDAVPQEKVVYSWYKNGKPARFSDRLLITRNDPELPPVTCSLEIIDLRFSDYGTYLCVATFQGAPIPDLSVEVNISAETVPPTISVPKGQSTITVREGSRAELQCEVRGKPKPPIIWSRVDKETPMPSGTMTVETYDGKLRLESVSRDMSGTYKCQTARYNSFNIRPREALVQLNVQFPPVVEPAFQDVRQGMGRSVTLRCTMLKGSPMKVATSVWRFNGTLLAQPPAEQQDCSELKVDSVSRETSGSYECSISNDVGVSACLFQVSAKAYSPEFYYDTPNPTLSQKQSKNYSYILQWTQKEPDAVDPILKYRLEVRQLAQRNTIQTFIPVQKMEKGLLLEHILPNLKVPQSYEVRLTPITSFGAGDMAARIIRYMEPINYPSPTDNTCRFEDEKICGFVQDKMDNFDWTRQNALTQNPKRTVNTGPPTDISGTPEGYYMFIEASRPRVTGDKARLISPLYNITAKYYCVSFYYHMYGKHIGSLNLLVRVRNKRAIDTQVWSLSGNRGNMWQQAHVPINPPGPFQIIFEGVRGTSYEGDIAIDDVTLKKGDCPRKPIGPNKAVALPGSGVSAQHGPCLCGPLTFFLYVLLR

>MDGA1_hs

MEVTCLLLLALIPFHCRGQGVYAPAQAQIVHAGQACVVKEDNISERVYTIREGDTLMLQCLVTGHPRPQVRWTKTAGSASDKFQETSVFNETLRIERIARTQGGRYYCKAENGVGVPAIKSIRVDVQYLDEPMLTVHQTVSDVRGNFYQEKTVFLRCTVNSNPPARFIWKRGSDTLSHSQDNGVDIYEPLYTQGETKVLKLKNLRPQDYASYTCQVSVRNVCGIPDKAITFRLTNTTAPPALKLSVNETLVVNPGENVTVQCLLTGGDPLPQLQWSHGPGPLPLGALAQGGTLSIPSVQARDSGYYNCTATNNVGNPAKKTVNLLVRSMKNATFQITPDVIKESENIQLGQDLKLSCHVDAVPQEKVTYQWFKNGKPARMSKRLLVTRNDPELPAVTSSLELIDLHFSDYGTYLCMASFPGAPVPDLSVEVNISSETVPPTISVPKGRAVVTVREGSPAELQCEVRGKPRPPVLWSRVDKEAALLPSGLPLEETPDGKLRLERVSRDMSGTYRCQTARYNGFNVRPREAQVQLNVQFPPEVEPSSQDVRQALGRPVLLRCSLLRGSPQRIASAVWRFKGQLLPPPPVVPAAAEAPDHAELRLDAVTRDSSGSYECSVSNDVGSAACLFQVSAKAYSPEFYFDTPNPTRSHKLSKNYSYVLQWTQREPDAVDPVLNYRLSIRQLNQHNAVVKAIPVRRVEKGQLLEYILTDLRVPHSYEVRLTPYTTFGAGDMASRIIHYTEPINSPNLSDNTCHFEDEKICGYTQDLTDNFDWTRQNALTQNPKRSPNTGPPTDISGTPEGYYMFIETSRPRELGDRARLVSPLYNASAKFYCVSFFYHMYGKHIGSLNLLVRSRNKGALDTHAWSLSGNKGNVWQQAHVPISPSGPFQIIFEGVRGPGYLGDIAIDDVTLKKGECPRKQTDPNKVVVMPGSGAPCQSSPQLWGPMAIFLLALQR

>MDGA1_md

MEVTCLLLLTLVPIYCKGQGVYAPAQAQIVHAGQACVVKEDNISERVYTIREGDTLVLQCLVTGHPRPQXXXXXXXXXXXXXXXXXXXXXXXXXXXXXXXXXXXXXXXXXXXXXXXXXXXXXXXXXXXLDEPVLTVHQTVSDVRGSFYQEKTVFLRCTVNSNPPARFIWKRGSSTLSHSQDDGVDIYEPLYTQGETKVLKLKNLRPQDYASYTCQVSVRNVCGIPDKAITFRLTNTTAPPALKLSVNETLVVNPGENVTVHCFLTGGDPQPQVLWSHGPGPLPLGSRAQGGTLTIPSVQAQDSGYYNCTAINNVGNPAKKTVNLLVRSMRNATFQITPDVIKESESIQLGQDLKLSCHVDAVPQEKVSYQWFKNGKLARVSDRLLVTRNDPELPAVTSSLELIDLRFSDYGTYLXXXXXXXXXXXXXXXXXXXXXXXXPPTISVPKGRSVVMVREGSSVELQCEVRGKPRPPVLWSRVDKEAGLLPSGAAMEETPDGKLRVERVSREMSGTYRCQTARYNGFNVRSREAQVQLNVQXXXXXXXXXXXXXXXXXXXXXXXXXXXXXXXXXXXXXXXXXXXXXXXXXXXXXXXXXXXXXXXXXXXXXXXXXXXXXXXXXXXXXXXXXXXXXXXXXKAYSPEFYYDTPNPTRSHKLSKNYSYVLQWTQKEPDAVDPVLSYRLDVRQLSLRTSITKPIPVRRVEKGQLLEYILTDLKVPHSYEVRLTPITTFGAGDMAARIIRYMEPTNSPNLSDNTCHFEDEKICGYTQDRADNFDWTRQNALTQNPKRSPNTGPPTDISGTPEGYYMFIETSRPRELGDRARLVSPLFNISAKLYCVSFFYHMYGKHIGSLNLLVRSRNKGALETHAWSLSGNRGNSWQQAHVPINPSGPFQXXXXXXXXXXXXXXXXXXXXXXXXXXXXXXXXXXVVMPGSGASCPPGPWLFGPLAIFLLAMLR

>MDGA1_mm

MEVTCLLLLALIPFHCRGQGVYAPAQAQIVHAGQACVVKEDNISERVYTIRESDTLVLQCLVTGHPRPQVRWTKTAGSASDKFQETSVFNETLRIERIARTQGGRYYCKAENGVGVPAIKSIRVDVQYLDEPVLTVHQTVSDVRGNFYQEKTVFLRCTVSSNPPARFIWKRGSDTLSHSQDNGVDIYEPLYTQGETKVLKLKNLRPQDYASYTCQVSVRNVCGIPDKAITFQLTNTTAPPALKLSVNETLVVNPGENVTVQCLLTGGDPLPQLHWSHGPGPLPLGALAQGGTLSIPSVQARDSGYYNCTATNNVGNPAKKTVNLLVRSLKNATFQITPDMIKESENIQLGQDLKLSCHVDAVPQEKVNYQWFKNGKPARTSKRLLVTRNDPELPAVTSSLELIDLHFSDYGTYLCMASFPGSPVPDLSIEVNISSETVPPTISVPKGRAVVTVREGSPAELQCEVRGKPRPPVLWSRVDKEAALLPSGLALEETPDGKLRLESVSRDMSGTYRCQTARYNGFNVRPREAQVQLTVHFPPEVEPSSQDVRQALGRPVLLRCSLLRGSPQRIASAVWRFKGQLLPPPPVLPAAAVETPDHAELRLDALTRDSSGNYECSVSNDVGSATCLFQVSAKAYSPEFYFDTPNPTRSHKLSKNYSYVLQWTQREPDAVDPVLNYRLSIRQLNQHNAMVKAIPVRRVEKGQLLEYILTDLRVPHSYEIRLTPYTTFGAGDMASRIIHYTEHNTCHFEDEKICGYTQDLTDNFDWTRQNALTQNPKRSPNTGPPTDISGTPEGYYMFIETSRPRELGDRARLVSPLYNASAKFYCVSFFYHMYGKHIGSLNLLVRSRNKGTLDTHAWSLSGNKGNVWQQAHVPINPSGPFQIIFEGVRGSGYLGDIAIDDVTLKKGECPRRQMDPNKVVVMPGSGAPRLSSLQLWGSMAIFLLALQR

>MDGA1_rn

MEVTCLLLLALIPFHCRGQGVYEAPAQAQIVHAGQACVVKEDNISERVYTIRESDTLVLQCLVTGHPRPQVRWTKTAGSASDKFQETSVFNETLRIERIARTQGGRYYCKAENGVGVPAIKSIRVDVQYLDEPVLTVHQTVSDVRGNFYQEKTVFLRCTVSSNPPARFIWKRGSDTLSHSQDNGVDIYEPLYTQGETKVLKLKNLRPQDYASYTCQVSVRNVCGIPDKSITFQLTNTTAPPTLKLSVNETLVVNPGENVTVQCLLTGGDPLPQLHWSHGPGPLPLGALAQGGTLSIPSVQARDSGYYNCTATNNVGNPAKKTVNLLVRSLKNATFQITPDMIKESENIQLGQDLKLSCHVDAVPQEKVNYQWFKNGKPARTSKRLLVTRNDPELPAVTSSLELIDLHFSDYGTYLCVASFPGSPVPDLSVEVNISSETVPPTISVPKGRAVVTVREGSPAELQCEVRGKPRPPVLWSRVDKEAALLPSGLALEETPDGKLRVERVSREMSGTYRCQTARYNGFNVRAREAQVQLTVHFPPEVEPSSQDVRQALGRPVLLRCSLLRGSPQRIASAVWRFKGQLLPPPPVLPAAAAEGPDHAELRLDLTRDSSGNYECSPEAPVTFPTCLFPLSAKAYSPEFYFDTPNPTRSHKLSKNYSYVLQWTQREPDAVDPVLNYRLSIRQLNQHNAMVKAIPVRRVEKGQLLEYTLTDLRVPHSYEIHLTPYTTFGAGDMASRVIHYTEPINSPSLSDNTCHFEDEKICGYTQDLTDNFDWTRQNALTQNPKRSPNTGPPTDISGTPEGYYMFIETSRPRELGDRARLVSPLYNASAKFYCVSFFYHMYGKHIGSLNLLVRSRNKGTLDTHAWSLSGNKGNVWQQAHVPINPSGPFQIIFEGVRGSGYLGDIAIDDVTLKKGECPRRQMDPNKVVVMPGSGAPRLSSLQLWGSMTIFLLALQR

>MDGA1_tg

MDMIWFLFLSLVPVYSRGQGVYAPAQAQIIHAGQACVVKEDNISERVYTIREGDTLVLQCLVTGHPRPQVRWTKTAGSASDKFQETSVLNETLRIEKIQRLQGGRYYCKAENGVGVPAIKSIRVDVQYLDEPVLTVHQTISDVRGSFYQEKTVFLRCTVNSNPPARFIWKRGAETLSHSQDNGVDIYEPLYTQGETKVLKLKNLRPQDYASYTCQVSVRNVCSIPDKSVTFQLTNTTAPPALKLSVNETLVVNPGDNITMQCSLTGGDPQPEVAWSHSPGPMPPNSLVQGGNLTIWRIRVEDSGYYNCTAINNVGNPAKKTVNLLVRSMKNATFQITPDVIKESETIQLGQDLKLSCHVDAVPQEKVVYSWYKNGKPARFSDRLLITRNDPELPPVTCSLEIIDLRFSDYGTYLCVATFQGAPIPDLSVEVNISSETVPPTISVPKGQSTITVREGSRAELQCEVRGKPKPPIIWSRVDKETPMPSGTMTMETYDGKLHLENVSREMSGTYRCQTARYNGFNIRPREALVQLNVQFPPVVEPAFQDVRQGTGHSVTLRCTMLKGSPMKVATSVWRFNGSLLAQPLAEQQDYSELKVDSVSRETSGSYECSISNDVGVSTCLFQVSAKAYSPEFYYDTPNPTLSQKQSKNYSYVLQWTQKEPDAVDPILKYRLEVRQLSQRNTIQTFIPVKQMEKGLLLEHILPNLKVPQSYEVRLTPITSFGAGDMSARVIRYMEPPSLPLPTDNTCRFEDEKICGFVQDKMDNFDWTRQNALTQNPKRTVNTGPPTDISGTPEGYYMFIEASRPRVTGDKARLISPLYNITAKYYCVSFYYHMYGKHIGSLNLLVRVRNKRAIDTQVWSLSGNRGNVWQQAHVPINPPGPFQIIFEGVRGTSYEGDIAIDDVTLKKGDCPRKPIGPNKAVALPGSSVPALHSPWLCGPLTFFLYVLLR

>MDGA_1_xt

MEILWLFLLSLIPRGNRGQGVYAPPQAQIIHAGQACVVKEDNISERVYTIREGDILVLQCLVTGHPRPQVRWTKTAGSASDKFQETSIYNETLYIEKVQRMQGGRYYCKAENGVGVPAIKSIRVDVQYLDEPILTIHQTISDVRGSFYQEKTVFLRCTVSSNPPARFIWKRGFEILSHNQDNGVDIYEPLYTQGETKVLKLKNLRPKDYANYTCQVSVRDVCGIPDKSITFQLTNRTAPPALRLSVNETLVVNPGDNVTIQCLLTGGDPPPKVVWSHNQNVVPSKGGNLTIWNVRPENSGYYNCTATNNVGNPAKKTVNLLVRSMKNATFQITPDVNKESENIQLGRDLKLSCHVDAVPQEKVVYSWFKNQRTVVHSRRRLEISRNDPDLPPDTSSLDFFDLRFSDYGTYMCVASFPGTPTPELSIEVNISAETVPPTLTVPTGQSIITVREGSRAELQCDARXKPKPPILWTRVDKEGPLPTGEMIAETYDGKLRLENVXXXXXXXXXXXXXXXXXXXXXXXXXXXXXXXXXAPVVEPKLREIRQPMHTGFTMRCDLLKGSPLKLATAVWRYNRNLLNVPPTEQMEYSELRINSLTQKTSGTYECTVSNDVGSDSCVFQVTGKALSPEFYYDTPSPIKVYKQFRNYSYLLQWTQKEPGAVDPITSYVLEYRQTTLRNTMSKNIPVGGIQKGQLQQYLLTDLKANQSYEVQLTPFTNFGAGDSASRIIRYTEHNTCRFDDDKMCGFVQDRKDNFDWTRQSMQTENPKRTANTGPRMDRSNTPEXXXXXXXXXXXXXXXXXXXXXXXXXXXRTTFCVSFYYHMYGRHIGNLNLLMRLKSKGTIDTQVWSQSGDRGNKWQHAMVPISATGQFQVVFEGVRGSGIEGDIAIDDVTVRKGECQWKQVPPSKDVMPPGSSTCKVLPTALHWAFFLFVLLR

>MDGA2_ac

XXXXXXXXXXXXXXXXXXXXXXXXAPPTVRIIHSGLACNIEEERYSERVYTIREGETLELTCLVTGHPRPQIRWTKTAGSASDRFQDSSVFNETLRISNIQRHQGGRYYCKAENGLGSPAIKSIRVDVYYLDDPIVTVHQSIGEAKEQFYYERTVFLRCVANSNPPVRYSWRRGLEVLLQGSDKGVEIYEPFFTQGETKILKLKNLRPQDYANYSCIASVRNVCNISDKMVSFRLSNKTASPSIKLLVDDPIVVNPGEAITLACVTTGGEPLPTLSWVRSVGSLPEKSSLKGGTLTIPAITSEDAGTYSCIANNNVGNPAKKSTNIIVRALKKGRFWITPDPYHKDDNIQIGREVKISCQVEAVPSEELTFSWFKNGRPLRSSERMVITQTDPDVSPGTTNLDIIDLKFTDFGTYTCVASLKGGGISDISIDVNISSSTVPPNLTVPQEKSPLVTREGDTVELQCQVTGKPKPIILWSRADKEVPMPDGSMQMESYDGILRIVNVSREMTGTYKCQTSQYNGFNVKPREALVQLIVQYPPAVEPAFLEIRQGQGRSVTMSCRVLRAYPTRVLTFEWRLGNKLLRTGQFDAQDSTEYIIRSLSRDNYGIYNCNIINEAGAGRCSFLVTGKAYAPEFYYDTYSPLWQNRARVYAYSLEWTQMNPDAVDRILTYHLGIRQAGQQRWWEQEIAVNGKIQKGELITYNLTELIKPEAYEVRLTPVTIFGDGDSTIRVIKYSAPMNPHLREFHCGFDDGNICLFTQDPKDNFDWTRQNIILRDTKYTPNTGPSADRTGSKDGFFMYIEASSPRKEGDRARLISPIFSIPPKNPYGATNTAYCFSFYYNMYGQHIGTLNVYLRLKSQTTLESPFWSSSGNKGQGWLQARVNIHPNTSFQVIFEGIRGRGIEGDIAVDDISIVEGECTKSESPDNHIPSGAGRRLPHIRIFPVFLLVSVLSHQR

>MDGA2_gg

MDVAIGLLGLLTVLLEGSSGQGVYAPPTVRIVHSGLACNIEEERYSERVYTIREGETLELTCLVTGHPRPQIRWTKTAGSASDRFQDSSVFNETLRIANIQRHQGGRYYCKAENGLGSPAIKSIRVDVYYLDDPIVTVHQSIGEAKEQFYYERTVFLRCVANSNPPVRYSWRRGQEVLLQGSDKGVEIYEPFFTQGETKILKLKNLRPQDYANYSCIASVRNVCSIPDKMVSFRLSNKTASPSIKLLVDDPIVVNPGEAVTLVCVTTGGEPAPSLTWVRSAGGLPEKTVLNGGTLTIPAIASEDAGTYSCVANNNVGNPAKKSTNIIVRALKKGRFWITPDPYHKDDNIQIGREVKISCQVEAVPSEELTFSWFKNGRPLRSSERMVITQTDPDVSPGTTNLDIIDLKFTDFGTYTCVASLKGGGISDISIDVNISSSTVPPNLTVPQEKSPLVTREGDTIELQCQVTGKPKPIILWSRADKEVAMPDGTMQMESYDGILRIVNVSREMTGTYRCQTSQYNGFNVKPREALVQLIVQYPPAVEPTFLEIRQGQGRSITMSCRVLRAYPTRVLTYEWRLGSKLLRTGQFDLQDSTEYTVSSLSRDSYGVYNCNIINEAGAGRCSFLVTGKAYAPEFYYDTYNPLWQNRPRVYSYSLQWTQMNPSAVDRILAYRLGIRQAGQQRWWEQEITVNGNIQKGELITYNLTELIKPEAYEVRLTPITRFGEGDSTIRVIKYSAPVNPHLREFHCGFEDGNICLFTQDDTDNFDWTKQSTATRDTKYTPNTGPNADRTGSKEGFYMYIETSRPRLEGEKARLVSPVFSVAPKNPYGATNTAYCFSFYYHMYGQHIGSLNVYLRLKGQTAIENPLWSSSGNKGQHWNQARVNINPPTSFQLIFEGIRGPGIEGDIAIDDVSIVEGECMKSDQPANNLRSGAVGTLEHIRLIPVTILMFVLSHQR

>MDGA2_hs

MDLLYGLVWLLTVLLEGISGQGVYAPPTVRIVHSGLACNIEEERYSERVYTIREGETLELTCLVTGHPRPQIRWTKTAGSASDRFQDSSVFNETLRITNIQRHQGGRYYCKAENGLGSPAIKSIRVDVYYLDDPVVTVHQSIGEAKEQFYYERTVFLRCVANSNPPVRYSWRRGQEVLLQGSDKGVEIYEPFFTQGETKILKLKNLRPQDYANYSCIASVRNVCNIPDKMVSFRLSNKTASPSIKLLVDDPIVVNPGEAITLVCVTTGGEPAPSLTWVRSFGTLPEKTVLNGGTLTIPAITSDDAGTYSCIANNNVGNPAKKSTNIIVRALKKGRFWITPDPYHKDDNIQIGREVKISCQVEAVPSEELTFSWFKNGRPLRSSERMVITQTDPDVSPGTTNLDIIDLKFTDFGTYTCVASLKGGGISDISIDVNISSSTVPPNLTVPQEKSPLVTREGDTIELQCQVTGKPKPIILWSRADKEVAMPDGSMQMESYDGTLRIVNVSREMSGMYRCQTSQYNGFNVKPREALVQLIVQYPPAVEPAFLEIRQGQDRSVTMSCRVLRAYPIRVLTYEWRLGNKLLRTGQFDSQEYTEYAVKSLSNENYGVYNCSIINEAGAGRCSFLVTGKAYAPEFYYDTYNPVWQNRHRVYSYSLQWTQMNPDAVDRIVAYRLGIRQAGQQRWWEQEIKINGNIQKGELITYNLTELIKPEAYEVRLTPLTKFGEGDSTIRVIKYSAPVNPHLREFHCGFEDGNICLFTQDDTDNFDWTKQSTATRNTKYTPNTGPNADRSGSKEGFYMYIETSRPRLEGEKARLLSPVFSIAPKNPYGPTNTAYCFSFFYHMYGQHIGVLNVYLRLKGQTTIENPLWSSSGNKGQRWNEAHVNIYPITSFQLIFEGIRGPGIEGDIAIDDVSIAEGECAKQDLATKNSVDGAVGILVHIWLFPIIVLISILSPRR

>MDGA2_md

XXXXXXXXXXXXXXXXXXXXXXXXXXXPPTVRIVHSGLACNIEEERYSERVYTIREGETLELTCLVTGHPRPQIRWTKTAGSASDRFQDSSVFNETLRITNIQRHQGGRYYCKAENGLGSPAIKSIRVDVYYLDDPVVTVHQSIGEAKEQFYYERTVFLRCVANSNPPVRYSWRRGQEVLLQGSDKGVEIYEPFFTQGETKILKLKNLRPQDYANYSCIASVRSVCNIPDKMVSFKLSNKTASPSIKLLVDDPIVVNPGEAITLVCVTTGGEPTPTLTWVKSIGNLPEKTVLNGGTLTIPAISSDDAGTYSCIANNNVGNPAKKSTSIIVRALKKGRFWITPDPYHKDDTIQIGREVKISCQVEAIPSEELIFSWFKNGRPLRSSERMVITQTDPDVSPGTTNLDIIDLKFTDFGTYTCVASLKGGGISDISIDVNISSSTVPPNLTVPQEKSPLVTREGDTIELQCQVTGKPKPIILWTRADKEVAMPDGSMQMESYDGTLRIVNVSREMSGMYRCQTSQYNGFNVTPREALVQLIVQYPPAVEPAFLEIRQGQDRSVTMSCRVLRAYPIRVLTYEWRLGNKLLRMGQFDSQEYTEYTLKDLSNENYGIYNCNIINEAGAGRCTYLVTGKAYAPEFYYDTYNPVWQNRHRVYSYSLQWTQMNPNAVDRIVAYRLGIRQAGQQRWWEQEIKISGNIQKGELITYNLTELIKPEAYEVRLTPLTKFGEGDSTIRIIKYSAPVNPHLREFHCGFEDGNICLFTQDDTDNFDWTKQSTATRNTKYTPNTGPNADRTGSKEGYYMYIETSRPRLEGEKARLLSPVFSIAPKNPYGATNTAYCFSFFYHMYGQHIGKINLFGADFRQTTIENPLWSSSGNKGQHWNEAHVNIYPITSFQLIFEGIRGPGIEGDIAIDDVSIAEGECAKQDLATKNSVDGAVGILVHIWLFPAVVLISILNPRR

>MDGA2_mm

MDLVYGLVWLLTVLLEGISGQGVYAPPTVRIVHSGLACNIEEERYSERVYTIREGETLELTCLVTGHPRPQIRWTKTAGSASDRFQDSSVFNETLRITNIQRHQGGRYYCKAENGLGSPAIKSIRVDVYYLDDPVVTVHQSIGEAKEQFYYERTVFLRCVANSNPPVRYSWRRGQEVLLQGSDKGVEIYEPFFTQGETKILKLKNLRPQDYANYSCIASVRNVCNIPDKMVSFRLSNKTASPSIKLLVDDPIVVNPGEAITLVCVTTGGEPTPSLTWVRSFGTLPEKIVLNGGTLTIPAITSDDAGTYSCIANNNVGNPAKKSTNIIVRALKKGRFWITPDPYHKDDNIQIGREVKISCQVEAVPSEELTFSWFKNGRPLRSSERMVITQTDPDVSPGTTNLDIIDLKFTDFGTYTCVASLKGGGISDISIDVNISSSTVPPNLTVPQEKSPLVTREGDTIELQCQVTGKPKPIILWSRADKEVAMPDGTMQMESYDGTLRIVNVSREMSGMYRCQTSQYNGFNVKPREALVQLIVQYPPAVEPAFLEIRQGQDRSVTMSCRVLRAYPIRVLTYEWRLGNKLLRTGQFDSQEYTEYPLKSLSNENYGVYNCSIINEAGAGRCSFLVTGKAYAPEFYYDTYNPVWQNRHRVYSYSLQWTQMNPDAVDRIVAYRLGIRQAGQQRWWEQEIKINGNIQKGELITYNLTELIKPEAYEVRLTPLTKFGEGDSTIRVIKYTGEFHCGFEDGNICLFTQDDTDNFDWTKQSTATRNTKYTPNTGPSADRSGSKEGFYMYIETSRPRLEGEKARLLSPVFSIAPKNPYGPTNSAYCFSFFYHMYGQHIGVLNVYLRLKGQTTIENPLWSSSGNKGQRWNEAHVNIYPITSFQLIFEGIRGPGIEGDIAIDDVSIAEGECAKQDLPTKNSVDGAVGILVHIWLFPVIILISILSPRR

>MDGA2_rn

MDLVYGLVWLLTVLLEGISGQGVYAPPTVRIVHSGLACNIEEERYSERVYTIREGETLELTCLVTGHPRPQIRWTKTAGSASDRFQDSSVFNETLRITSIQRHQGGRYYCKAENGLGSPAIKSIRVDVYYLDDPVVTVHQSIGEAKEQFYYERTVFLRCVANSNPPVRYSWRRGQEVLLQGSDKGVEIYEPFFTQGETKILKLKNLRPQDYANYSCIASVRNVCNIPDKMVSFRLSNKTASPSIKLLVDDPIVVNPGEAITLVCVTTGGEPMPSLTWVRSFGTLPEKIVLNGGTLTIPAITSDDAGTYSCIANNNVGNPAKKSTNIIVRALKKGRFWITPDPYHKDDNIQIGREVKISCQVEAVPSEELTFSWFKNGRPLRSSERMVITQTDPDVSPGTTNLDIIDLKFTDFGTYTCVASLKGGGISDISIDVNISSSTVPPNLTVPQEKSPLVTREGDTIELQCQVTGKPKPIILWSRADKEVAMPDGTMQMESYDGTLRIVNVSREMSGMYRCQTSQYNGFNVKPREALVQLIVQYPPAVEPAFLEIRQGQDRSVTMSCRVLRAYPIRVLTYEWRLGNKLLRTGQFDSQEYTEYPLKSLSNENYGVYNCSIINEAGAGRCSFLVTGKAYAPEFYYDTYNPVWQNRHRVYSYSLQWTQMNPDAVDRIVAYRLGIRQAGQQRWWEQEIKINGNIQKGELITYNLTELIKPEAYEVRLTPLTKFGEGDSTIRVIKYTGEFHCGFEDGNICLFTQDDTDNFDWTKQSTATRNTKYTPNTGPNADRSGSKEGFYMYIETSRPRLEGEKARLLSPVFSIAPKNPYGPTNSAYCFSFFYHMYGQHIGVLNVYLRLKGQTTIENPLWSSSGNKGQRWNEAHVNIYPITSFQLIFEGIRGPGIEGDIAIDDVSIAEGECAKQDLPTKNSVDGAVGILVHIWLFPVIILISILSPRR

>MDGA2_tg

XXXXXXXXXXXXXXXXXXXXXXXXXPPTVRIVHSGLACNIEEERYSERVYTIREGETLELTCLVTGHPRPQIRWTKTAGSASDRFQDSSVFNETLRISNIQRHQGGRYYCKAENGLGSPAIKSIRVDVYYLDDPIVTVHQSIGEAKEQFYYEKTVFLRCVANSNPPVRYSWRRGQEVLLQGSDKGVEIYEPFFTQGETKILKLKNLRPQDYANYSCIASVRNVCSIPDKMVSFRLSNKTASPSIKLLVDDPIVVNPGEAITLVCVTTGGEPVPSLTWVRSAGVLPDKTVLNGGTLTIPAITSEDSGTYSCIANNNVGNPAKKSTNIIVRALKKGRFWITPDPYHKDDNIQIGREVKISCQVEAIPSEELTFSWFKNGRPLRSSERMVITQTDPDVSPGTTNLDIIDLKFTDFGTYTCVASLKGGGISDISIDVNISSSTVPPNLTVPQEKSPLVTREGDTIELQCQVTGKPKPIILWSRADKEVAMPDGALQTESYDGILRIVNVSREMTGTYRCQTSQYNGFNVKPREALVQLIVQXXXXXXXXXXXXXXXXXXXXXXXXXXXXXXXXXXXXXXXXXXXRVLRTGQFDLQDSTEYTVSSLSRDSYGVYNCNIINEAGAGRCSFLVTGKAYAPEFYYDTYNPLWQNRPRVYSYSLQWTQMNPGAVDRILAYRLGIRQAGQQRWWEQEITVNGNIQKGELITYNLTELIKPEAYEVRLTPITRFGEGDSTIRVIKYSAPVNPHLRKFHWGFEDGNICLFTQNDTNNFDWTKQSTATRDTKYTPNTGPNADRTGSKEGFYMYIETSRPRLEGEKARLVSPVFNVAPKNPYGATNTAYCFSFYYHMYGQHIGSLNVYLRLKGQTAIENPLWSSSGNKGQHWNQARVNINPPTSFQLIFEGIRGPGIEGDIAIDDVSIVEGECMKSDQPANNLRSGAVGPLAHIRLLPLLILMSVLSHQR

>MDGA2_xt

MDLGYGVLWLLTVLLEGISCQGVYAPPTVRIIHSGLACNIEEERYSERVYTIREGETLELTCLVTGHPRPQIRWTKTAGSASDRFQDSSVFNETLRIENIQRHQGGRYYCKAENGLGSPAIKSIRVDVYYLDDPIVTVHQSIGEAKEQFYYERTVFLRCIANSNPPVRYSWKRGEEILLQGSDKGVEIYEPFFTQGETKILKLKNLRPQDYANYSCIASVRNVCNIPDKIVSFKLSNRTASPSIKLLVDDPIVVNPGEAITLVCVTTGGEPTPTLMWVRSTGTLPEKTVLKGGTLTIPAITSEDAGTYSCIANNNVGNPAKKSTSIIVRALKKGRFWITPDPYHKDDNIQIGREVKISCQVEAVPSEELTFSWFKNGRPLRSSERMVITQTDPDVSPGTTNLDIIDLKFTDFGTYTCVASLKGGGISDISIDVNISSSTVPPNLTVPKGKSPMVAREGDTIELQCQISGKPKPIIMWSRADKDVPMPDGSMQMESYDGILRIVNVSREMTGTYRCQTSQYNGFNVKPRDALVQLIVQYPPAVEPVFQEIRQGLGHSVTMNCRVLRAYPTKVLMYEWRLGSKLLEKGQFDSQDYTELTVNILAKEHYGTYNCSIINEAGSGRCSFLVTGKAYPPEFYYDTYNPLWQNKAQIYSYKLQWTQMNPDAVDRILAYRLGIRQAGLQRWWEREISVNGIIKKGELMTNNLTELVRPEPYEVRLTPINRFGEGDSAIRIIKYSGNSSAVVLTGEFHCGFEESNICLFTQDDTDIFDWTKQSASTRDTRYTPNTGPTTDAGGSKHGFYMYIETSRPRMDGEKARLLSPIFNIAQKNPYGSSNTVYCFSFFYHMYGKHIGALNIFLRLKGQTSTDIPLWSAKGNKGEQWKQTHLNIHPTSSFQLIFEGIRGDGIEGDIAIDDISIMEGECSIIDQPDTSTGNGTFIHIWLYPVFLFLSVLSHQR

>MDGA_ci

XXXXXXXXXXXXXXXXXXXXXXXXXXXXXXXXXXXXXXXXXXXXXXESIYNIKEYDNVVLTCIVSGHPLPRIRWSMSGGALDNKKLDLQSPILRIYNITRRESCRAVCRATNGIERPAVASKRINVLYLDRPVLTAHLTNKGKIPSTSIWFENTIYLHCSVTSNPPASFSWLLNGQRLLVRRVDPYNKDISTVILKIKDAGSLNIGNYQCHTSLDPWLRKYGVSNTKDNFTYTTNIAPPRIQLSTPSLIVVNTGDRVRMQCNVTGGIPVSKLDWATTNGVFSDRVVFSKLRNELIVDSVRVDDSADYFCIANNQVGIPVQKKTTLIVRNIGPMTYWLTPDPLRDDRSFAVNTDIKLTCHADAAPYSELTYFWYKKQCNRVEISFLQKQISRSNNVQLEIRSLNEDDYGTYVCSARLAPNGTESQFNLDTSNFDFAAPAKIFASNSLVVVTEGSSALLQCRTSGKPKPLVLWFRNENTTLPSGEMQEVTQDGILTINQTSRDHADLYTCTTARFNGFSKLSKTQSEAVVELQVQYAPTVEPSYVEIRRSLGSDVTLSCFVSDASPMAPITYVWKKENVIQSTSFAGQQLVIQSIGPTDYGVYVCEVTNVVGKAQCVLNVTAFAFPPEFLFTEFGEFQENPTGTIFPQTGTDSDTFIYLLSWTQKHPKKPDKINSYRIKLNCSIFNSYCMTMLSIYFKDALSYGELLTYQMRNLVKGMYEVEVTPKTIFGYGDSSSRQIMVRDRNLSYFCKFEDSDLCGFIQEKHPDMDSIDWTKNSAKTQGRRTKNTGPVADHSTLSREGAYMYIEASVTKKNDRARLLSPVFFDKSSFQQCLKFWYHMKGRHIGVLRVLVKKRGENETEIWRLSGAQGNTWRTGLVTITHTTPYQLIFEAIMTYDNGYQGDIAIDDILLADGKCSYRPTTTXXXXXXXXXXXXXXXXXXXXXXXXXXX
